# Systematic Multi‐Level Analyses Decode the Arthritis‐Neurodegeneration Axis With In Vivo Validation

**DOI:** 10.1002/advs.76930

**Published:** 2026-08-05

**Authors:** Jinwen Wang, Wenhui Xie, Lei Yang, Zhengrong Wang, Yi Huang, Peng Huang, Yuan Liu, Jun Hu

**Affiliations:** ^1^ Department of Orthopedics The First Affiliated Hospital with Nanjing Medical University Nanjing China; ^2^ Department of Biostatistics School of Public Health Nanjing Medical University Nanjing China; ^3^ Department of Infectious Diseases The First Affiliated Hospital with Nanjing Medical University Nanjing China; ^4^ Department of Epidemiology Center For Global Health School of Public Health Nanjing Medical University Nanjing China

**Keywords:** comorbidity, neurodegenerative diseases, osteoarthritis, parkinson's disease, rheumatoid arthritis, ring finger protein 40

## Abstract

Arthritis may influence neurodegenerative risk, but directionality and mediators remain unclear. This study integrates population survival analysis, Mendelian randomization, transcriptomic mapping, and mouse perturbation to map osteoarthritis (OA)/rheumatoid arthritis (RA) links with five neurodegenerative outcomes and prioritize mediators. In 310 162 European‐ancestry UK Biobank participants, Cox models associate OA with higher risks of Alzheimer's disease (AD; hazard ratio: 1.13, 95% confidence interval: 1.04–1.22), Parkinson's disease (PD; 1.10, 1.00–1.21), and disorders of autonomic nervous system (DANS; 1.36, 1.06–1.74), and RA with higher AD risk (1.37, 1.13–1.67) (all *p* < 0.05), but not incident PD. Mendelian randomization prioritizes a modest protective genetic effect of RA on PD (odds ratio: 0.93, 0.88–0.99; *p* = 0.015), without reverse causation. Transcriptome‐wide association and colocalization analyses identify shared RA–PD genes and prioritize Ring Finger Protein 40 (*RNF40*). In a collagen‐induced arthritis (CIA) and 1‐methyl‐4‐phenyl‐1,2,3,6‐tetrahydropyridine (MPTP) mouse model, CIA attenuates dopaminergic injury, whereas systemic *Rnf40* knockdown alleviates arthritis but exacerbates Parkinsonian pathology. Endogenous RNF40 is induced in arthritic joints but remains stable in midbrain. These cross‐layer data define arthritis–neurodegeneration connections and nominate RNF40 as a context‐dependent joint–brain candidate linking inflammatory arthritis with dopaminergic vulnerability.

## Graphical abstract

Arthritis and neurodegeneration are usually studied as separate disorders, but this study connects them through population evidence, genetic inference, transcriptomic mapping, and mouse models. It highlights RNF40 as a context‐dependent joint‐brain candidate, induced in inflammatory joints yet functionally linked to dopamine‐neuron vulnerability, revealing a new route forimmune‐neural crosstalk in arthritis and Parkinsonian disease.

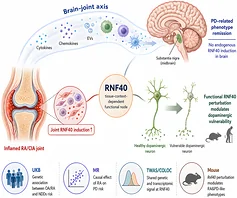

## 1 Introduction

Arthritis and neurodegenerative diseases (NDDs) affect distinct organ systems, yet they converge on immune programs that can couple peripheral inflammation to neural injury [1, 2, 3]. Whether arthritis causally shifts NDD risk remains central to mapping immune neural crosstalk and defining tractable targets for cross‐system intervention [4, 5]. Rheumatoid arthritis (RA) and osteoarthritis (OA) impose substantial global burden (0.24% to 2% and 7.6% prevalence, respectively) [6, 7], while Alzheimer's disease (AD), Parkinson's disease (PD) and multiple sclerosis (MS) drive progressive cognitive and motor decline and are projected to rise markedly through 2050 [8, 9, 10, 11]. Even modest arthritis‐related risk shifts could therefore translate into sizeable population‐level neurodegenerative burden. Mechanistically, chronic cytokine signaling, systemic immune remodeling and immune cell trafficking may disrupt blood‐brain barrier integrity and amplify neuroinflammation [12, 13]. However, current evidence is dominated by non‐randomized designs, leaving residual confounding and reverse causation difficult to exclude and offering limited molecular specificity for action.

Cohort studies and Mendelian randomization (MR) have linked arthritis to outcomes such as AD and PD, with some suggesting concordant causal directions [14, 15, 16, 17, 18, 19]. Yet the field still lacks a closed evidentiary chain that links population associations to causal genetic inference, localizes convergent pathways and demonstrates in vivo function. Observational and quasi‐experimental designs remain sensitive to unmeasured confounding, treatment era effects and cross cohort heterogeneity [20, 21]. MR reduces some biases under explicit assumptions but can be limited by weak instruments, horizontal pleiotropy, phenotype misclassification and sample overlap, such that no single analytic route is likely to close the loop from causation to mechanism to function [22, 23].

To close this gap, we apply a stepwise triangulation framework anchored in the UK Biobank (UKB) [24]. We first quantify associations of RA and OA with prespecified neurodegenerative phenotypes and test key effect modifiers. We then interrogate causal directionality and shared genetic architecture for AD, PD and MS using bidirectional MR, and prioritize convergent, tissue‐contextualized candidates using expression‐informed analyses and colocalization. Finally, we test the prioritized node in murine models to link population level signals to mechanistic and phenotypic consequence. By expanding phenotypic scope in UKB and coupling causal inference to experimental validation, this study aims to delineate actionable inflammation–neural nodes that can inform drug repurposing, risk stratification, and precision prevention.

## 2 Results

### 2.1 Clinical Characteristics of the Participants

Baseline characteristics of the study population are summarized in Table S1. Among the 310,162 participants (mean age 56.65±8.02 years; 53.7% female), we identified 81,802 cases of OA, 7,848 of RA, 2,631 of AD, 2,240 of PD, 361 of MS, 363 of DANS, and 459 of SMA (Figure 1).

**FIGURE 1:**
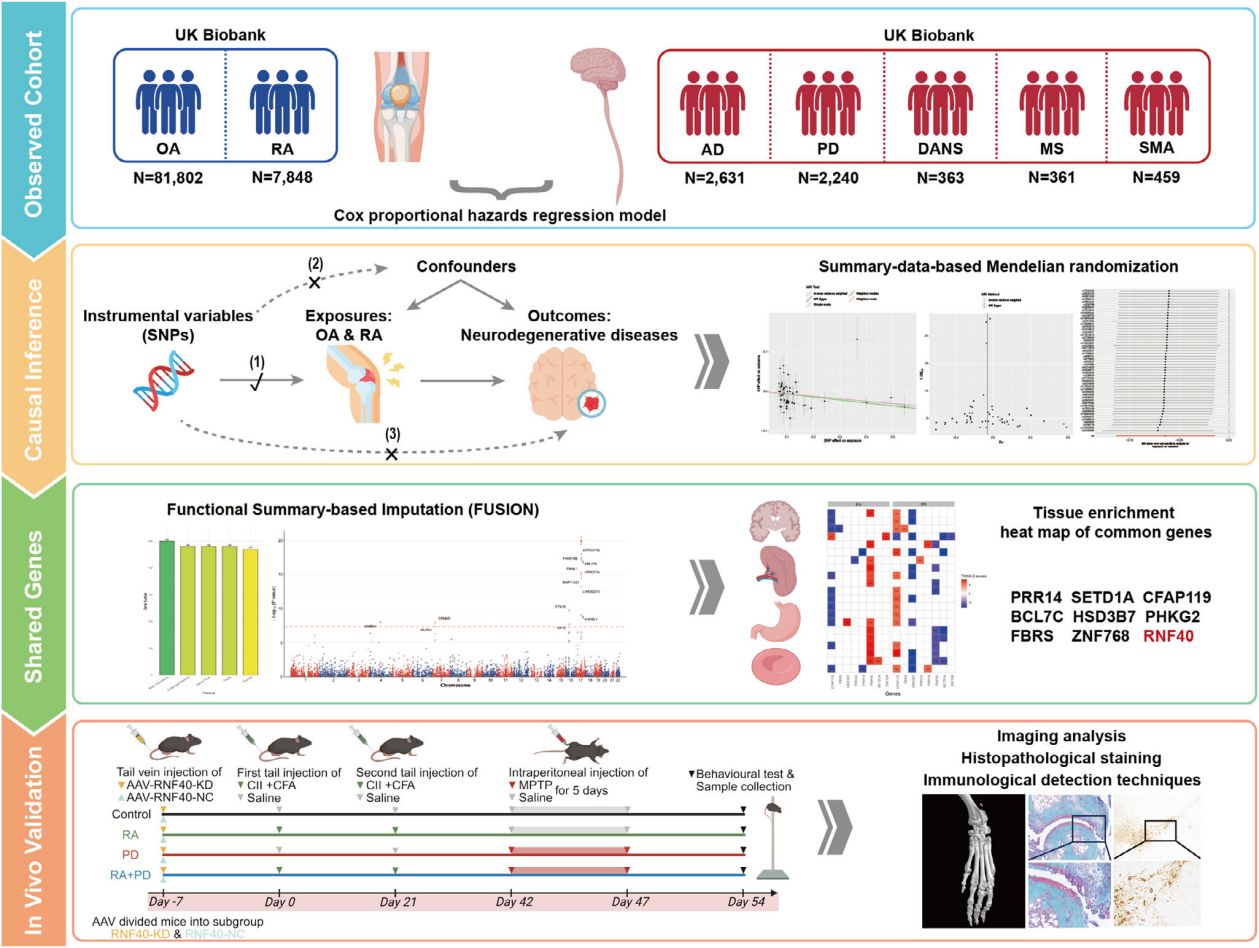
Flowchart of the study. A multi‐level framework integrating UK Biobank observational analyses, bidirectional Mendelian randomization, TWAS, colocalization analyses, and CIA–MPTP experimental validation was used to investigate the arthritis–neurodegeneration axis and prioritize RNF40 as a candidate context‐dependent mediator linking arthritis‐like inflammation with parkinsonian neurodegeneration. OA, Osteoarthritis; RA, Rheumatoid Arthritis; AD, Alzheimer's disease; PD, Parkinson's disease; DANS, Disorders of autonomic nervous system; MS, Multiple sclerosis; SMA, Spinal muscular atrophy and related syndromes; TWAS, transcriptome‐wide association study; CIA, collagen‐induced arthritis; MPTP, 1‐methyl‐4‐phenyl‐1,2,3,6‐tetrahydropyridine; SNP, single nucleotide polymorphism. Schematic elements were created in BioRender. Wang, J. (2026) https://BioRender.com/q3gwwmx.

### 2.2 Prospective Associations Between OA/RA and Incident Neurodegenerative Outcomes

In multivariable Cox proportional hazards models, OA was associated with increased risks of incident AD (HR 1.13, 95% CI 1.04–1.22; *p* = 0.006), PD (HR 1.10, 95% CI 1.00–1.21; *p* = 0.049), and DANS (HR 1.36, 95% CI 1.06–1.74; *p* = 0.015). The OA–PD association was modest and should be interpreted cautiously given its borderline statistical significance. For RA, a significant association was observed with incident AD (HR 1.37, 95% CI 1.13–1.67; *p* = 0.001), whereas RA was not significantly associated with incident PD, DANS, MS, or SMA (Table 1). Supplementary logistic regression analyses were conducted to compare binary‐outcome OR estimates with time‐to‐event HR estimates (Figure S1, Tables S2 and S3). In the medication‐restricted sensitivity analysis, we further excluded participants with recorded use of DMARDs, biologics/JAK inhibitors, glucocorticoids, or NSAIDs. The associations of OA with increased risks of AD and DANS remained robust, and the association between RA and AD was also consistent (Table S4).

**TABLE 1:** Cox proportional hazards analyses of OA/RA and incident neurodegenerative outcomes in UK Biobank.

	OA ^a)^ (n = 81,802)				RA ^b)^ (n = 7,848)			
	Crude model ^h)^ (Model 1)		Fully adjusted model ^i)^ (Model 2)		Crude model (Model 1)		Fully adjusted model (Model 2)	
	HR ^j)^ (95%CI)	*P*	HR (95%CI)	*P*	HR (95%CI)	*P*	HR (95%CI)	*P*
AD ^c)^	1.19 (1.10, 1.29)	<0.001	1.13 (1.04, 1.22)	0.006	1.53 (1.26, 1.86)	<0.001	1.37 (1.13, 1.67)	0.001
PD ^d)^	1.12 (1.02, 1.23)	0.017	1.10 (1.00, 1.21)	0.049	1.22 (0.95, 1.58)	0.127	1.18 (0.91, 1.53)	0.201
DANS ^e)^	1.39 (1.09, 1.76)	0.007	1.36 (1.06, 1.74)	0.015	1.91 (1.10, 3.34)	0.023	1.75 (0.99, 3.09)	0.054
MS ^f)^	1.06 (0.81, 1.40)	0.667	0.96 (0.72, 1.27)	0.768	0.96 (0.43, 2.15)	0.917	0.68 (0.28, 1.65)	0.390
SMA ^g)^	1.15 (0.93, 1.42)	0.212	1.14 (0.92, 1.42)	0.228	1.39 (0.80, 2.41)	0.248	1.39 (0.79, 2.44)	0.250

^a)^ OA, Osteoarthritis;

^b)^ RA, Rheumatoid Arthritis;

^c)^ AD, Alzheimer's disease;

^d)^ PD, Parkinson's disease;

^e)^ DANS, Disorders of autonomic nervous system;

^f)^ MS, Multiple sclerosis;

^g)^ SMA, Spinal muscular atrophy and related syndromes;

^h)^ Crude model: adjusting for Age, Sex;

^i)^ Fully adjusted model: adjusting for Age, Sex, TDI, Education, BMI, Smoking, Alcohol, Cancer, CHD, Diabetes, Fracture History and Medication history;

^j)^ HR, hazard ratios.

In subgroup analyses (Figure 2), the OA‐AD association was directionally consistent across age strata and remained evident in men, overweight individuals, and participants without T2D. The OA‐DANS association appeared more pronounced in younger participants, women, and individuals with higher BMI. For RA, the association with AD was more apparent in older participants, women, individuals with higher BMI, never‐smokers, and particularly those without T2D.

**FIGURE 2:**
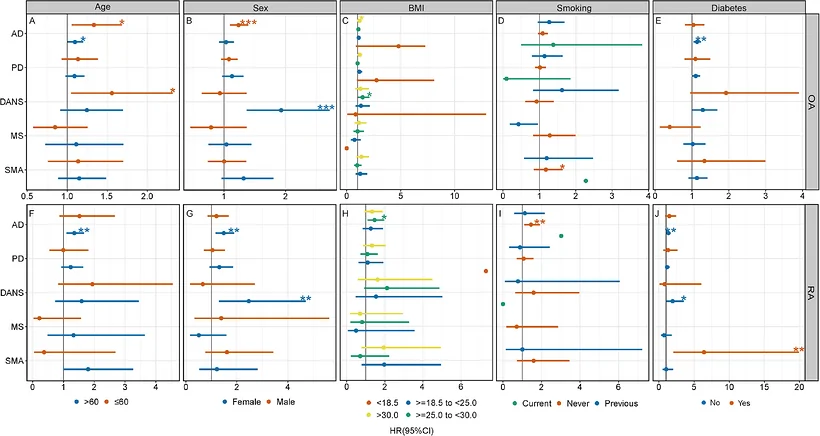
Forest plot of Cox hazard ratios for associations of OA/RA with incident neurodegenerative outcomes. (A) HRs associated with age for OA patients with neurodegenerative outcomes. (B) HRs associated with sex for OA patients with neurodegenerative outcomes. (C‐E) HRs associated with BMI, smoking status and diabetes for OA patients with neurodegenerative outcomes. (F‐J) HRs associated with age, sex, BMI, smoking status and diabetes for RA patients with neurodegenerative outcomes. All analyses were performed using Cox proportional hazards regression model.

### 2.3 Bidirectional MR and Sensitivity Analyses

To delineate causal relationships between OA, RA, and three NDDs (AD, PD and MS), we performed bidirectional two‐sample MR (Table 2, Figure 3, Figures S2–S5).

**TABLE 2:** Bidirectional Mendelian randomization analyses of OA/RA and three neurodegenerative diseases.

		IVW ^a)^ (main analysis)		MR‐Egger		Weighted median		Simple mode		Weighted mode	
Exposure	Outcome	OR ^b)^ (95% CI)	*P*	OR (95% CI)	*P*	OR (95% CI)	*P*	OR (95% CI)	*P*	OR (95% CI)	*P*
*The forward MR* ^c)^ *analyses*											
OA ^d)^	AD ^f)^	1.03 (0.91, 1.15)	0.677	0.86 (0.47, 1.58)	0.633	1.01 (0.88, 1.16)	0.877	1.03 (0.77, 1.39)	0.825	1.02 (0.78, 1.33)	0.902
	PD ^g)^	1.16 (0.92, 1.45)	0.206	0.60 (0.18, 2.00)	0.410	1.20 (0.88, 1.66)	0.241	0.92 (0.49, 1.73)	0.791	1.60 (0.91, 2.81)	0.119
	MS ^h)^	0.91 (0.60, 1.39)	0.669	1.34 (0.09, 19.34)	0.833	1.13 (0.81, 1.58)	0.470	1.24 (0.74, 2.10)	0.426	1.24 (0.72, 2.15)	0.447
RA ^e)^	AD	1.00 (0.98, 1.02)	0.945	1.00 (0.97, 1.04)	0.866	1.01 (0.98, 1.03)	0.541	1.00 (0.99, 1.06)	0.992	1.00 (0.98, 1.03)	0.913
	PD	0.94 (0.89, 0.99)	0.012	0.94 (0.86, 1.02)	0.163	0.93 (0.88, 0.99)	0.015	0.93 (0.83, 1.03)	0.167	0.94 (0.89, 0.99)	0.025
	MS	1.03 (0.88, 1.21)	0.713	0.87 (0.67, 1.14)	0.327	1.00 (0.92, 1.10)	0.952	0.88 (0.76, 1.03)	0.110	0.96 (0.55, 1.67)	0.874
*The reverse MR analyses*											
AD	OA	0.99 (0.96, 1.02)	0.448	0.98 (0.92, 1.05)	0.656	0.98 (0.94, 1.02)	0.330	0.96 (0.88, 1.06)	0.435	0.95 (0.90, 1.01)	0.135
	RA	1.00 (0.91, 1.11)	0.969	0.85 (0.67, 1.08)	0.198	0.99 (0.89, 1.09)	0.762	0.96 (0.80, 1.16)	0.677	0.97 (0.86, 1.09)	0.579
PD	OA	0.99 (0.96, 1.02)	0.603	0.94 (0.87, 1.02)	0.173	0.99 (0.95, 1.02)	0.469	1.00 (0.94, 1.06)	0.910	0.98 (0.94, 1.03)	0.498
	RA	1.02 (0.95, 1.08)	0.656	1.12 (0.94, 1.33)	0.220	1.05 (0.98, 1.13)	0.170	1.07 (0.94, 1.21)	0.328	1.07 (0.97, 1.18)	0.174
MS	OA	1.00 (0.99, 1.02)	0.660	1.03 (0.99, 1.09)	0.361	1.00 (0.98, 1.02)	0.776	0.99 (0.94, 1.04)	0.690	0.99 (0.94, 1.03)	0.584
	RA	0.97 (0.89, 1.06)	0.519	0.81 (0.70, 0.93)	0.004	0.95 (0.90, 0.99)	0.026	1.22 (1.00, 1.50)	0.058	0.93 (0.84, 1.04)	0.198

^a)^ IVW: inverse variance weighted;

^b)^ OR, odds ratio;

^c)^ MR, Mendelian randomization;

^d)^ OA, Osteoarthritis;

^e)^ RA, Rheumatoid arthritis;

^f)^ AD, Alzheimer's disease;

^g)^ PD, Parkinson's disease;

^h)^ MS, Multiple sclerosis.

**FIGURE 3:**
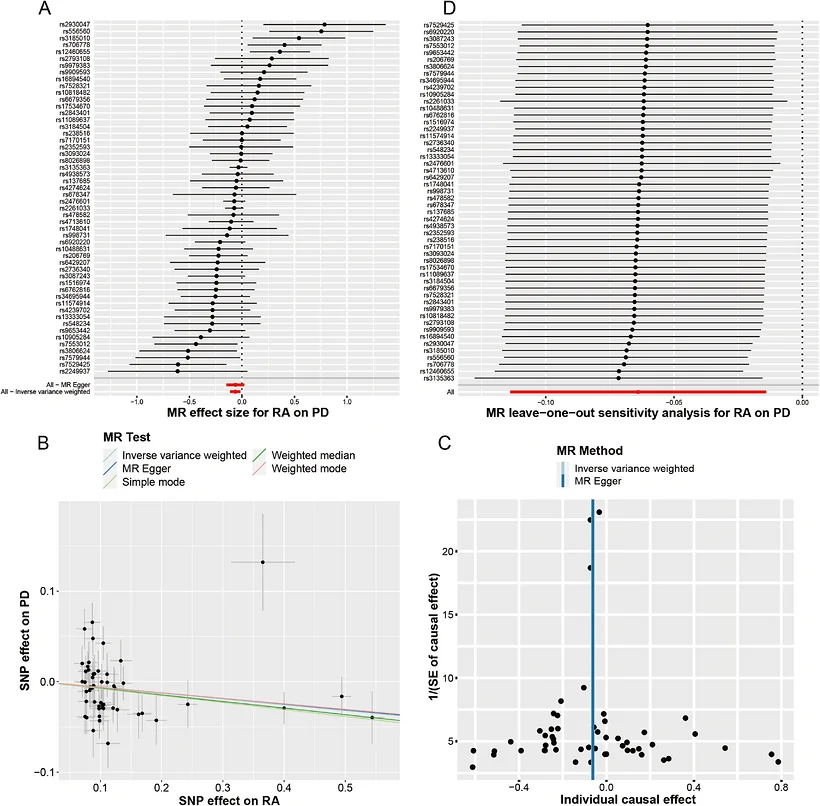
Summary of MR analysis between RA and PD. (A) Forest plot of the causal effects of RA associated SNPs on PD. (B) Scatter plot showed causal effects of RA on PD. The slopes of line represent the causal effect of each method, respectively. (C) Funnel plot of the causal effect of RA on PD. (D) Leave‐one‐out analysis plots for RA on PD. MR, Mendelian randomization; SNP, single nucleotide polymorphism; RA, Rheumatoid arthritis; PD, Parkinson's disease.

Results of the MR analysis for the causal effect of RA on PD are shown in Table 2, Table S5 and Figure 3A,B. There was evidence of heterogeneity across instruments (Cochran's Q = 88.98, *p* = 0.001; Figure 3C) but no indication of directional pleiotropy (MR‐Egger intercept = −0.0003, *p* = 0.958). Under this pattern, we emphasized the weighted median estimate, which supported a protective causal effect of RA on PD (OR 0.93, 95% CI 0.88–0.99; *p* = 0.015). Leave‐one‐out analysis confirmed this signal was not driven by individual outlier SNPs (Figure 3D). Reverse MR provided no evidence for a causal effect of PD on RA (OR 1.02, 95% CI 0.95–1.08; *p* = 0.656) (Table 2, Figure S2).

For the MS–RA pair, MR estimates are reported in Table 2, Table S5 and Figure S3A, B. In the analysis of MS as the exposure, there was substantial heterogeneity (Q = 948.73, *p* < 0.001; Figure S3C) and clear evidence of horizontal pleiotropy (MR‐Egger intercept = 0.039, *p* = 0.002). Under these conditions, the MR‐Egger estimator was considered more appropriate and suggested a protective effect of genetic liability to MS on RA (OR 0.81, 95% CI 0.70–0.93; *p* = 0.004). However, leave‐one‐out sensitivity testing revealed this estimate was vulnerable to single‐SNP omission (Figure S3D, Table S6), precluding definitive causal claims. Reverse MR yielded no causal effect of RA on MS (OR 1.00, 95% CI 0.92–1.10; *p* = 0.952) (Table 2, Figure S4).

MR models evaluating OA with AD, PD, and MS, as well as RA with AD, failed to identify consistent causal effects across primary and sensitivity estimators (Table 2, Figure S5). Consequently, the protective effect of RA on PD emerged as the singular robust causal relationship.

### 2.4 TWAS of the RA–PD Axis

Given the Mendelian randomization evidence prioritizing the RA–PD relationship, we focused downstream transcriptomic analyses on the RA–PD axis. Using the FUSION pipeline, we integrated GWAS summary statistics for RA and PD with gene expression prediction models for 49 human tissues. Applying a study‐wide significance threshold, we identified 304 genes significantly associated with RA and 147 with PD.

For RA, the largest numbers of significant genes were observed in spleen, whole blood, thyroid, esophagus mucosa and tibial nerve, whereas PD signals were enriched in esophagus mucosa, brain cerebellum, tibial nerve, thyroid and testis (Figure 4A,B). Focusing on the tibial nerve—a tissue with strong involvement for both traits—we identified 41 RA‐associated genes and 48 PD‐associated genes (Figure 4C,D, Tables S7 and S8).

**FIGURE 4:**
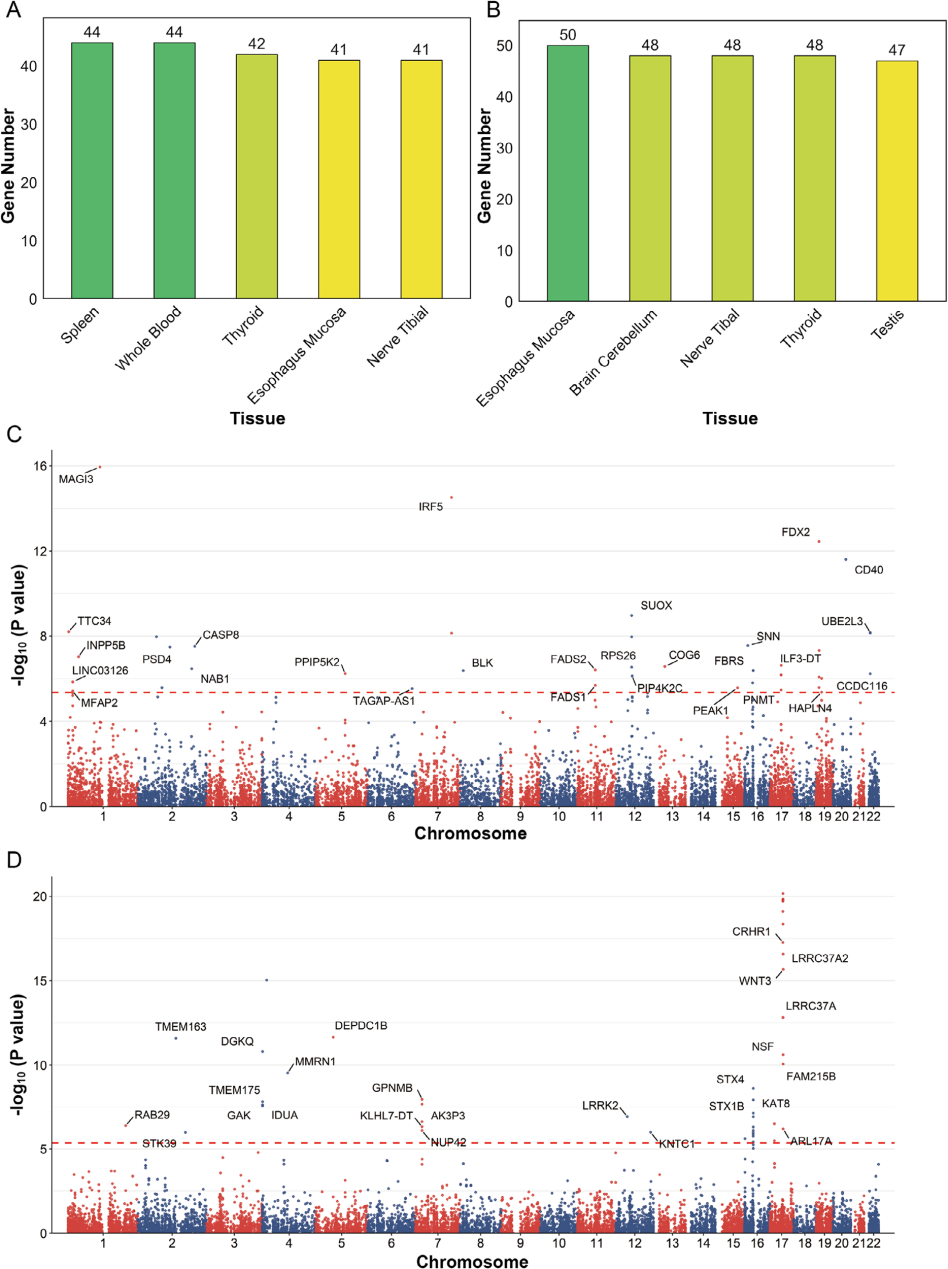
Transcriptome‐wide association study (TWAS) identifies genes associated with rheumatoid arthritis and Parkinson's disease. (A) Top five tissues in Rheumatoid arthritis with the highest number of TWAS‐significant genes. (B) Top five tissues in Parkinson's disease with the highest number of TWAS‐significant genes. (C) Genome‐wide Manhattan plot of transcriptome‐wide association study results for RA‐related genes in the tibial nerve. (D) Genome‐wide Manhattan plot of transcriptome‐wide association study results for PD‐related genes in the tibial nerve.

Comparative analysis identified eight genes reaching transcriptome‐wide significance for both traits: *PRR14*, *CFAP119*, *HSD3B7*, *RNF40*, *SETD*1A, *PHKG2*, *ZNF768*, and *FBRS* (Figure 5). Several exhibited tissue‐dependent directional heterogeneity (e.g., *CFAP119*, *FBRS* and five other genes). Among the eight shared genes, *RNF40* uniquely displayed cross‐trait, directionally consistent TWAS associations across multiple tissues, distinguishing it from the remaining candidates, which showed more restricted or directionally heterogeneous patterns. Specifically, *RNF40* expression was positively associated with RA but negatively associated with PD in key peripheral and neuro‐relevant regions, including esophagus muscularis, transverse colon, substantia nigra, cerebellum, cerebellar hemisphere, basal ganglia (caudate nucleus), adrenal gland and visceral adipose tissue (Figure 5). This robust cross‐tissue alignment prioritized *RNF40* as a candidate comorbidity gene along the RA–PD axis, motivating subsequent functional validation in vivo.

**FIGURE 5:**
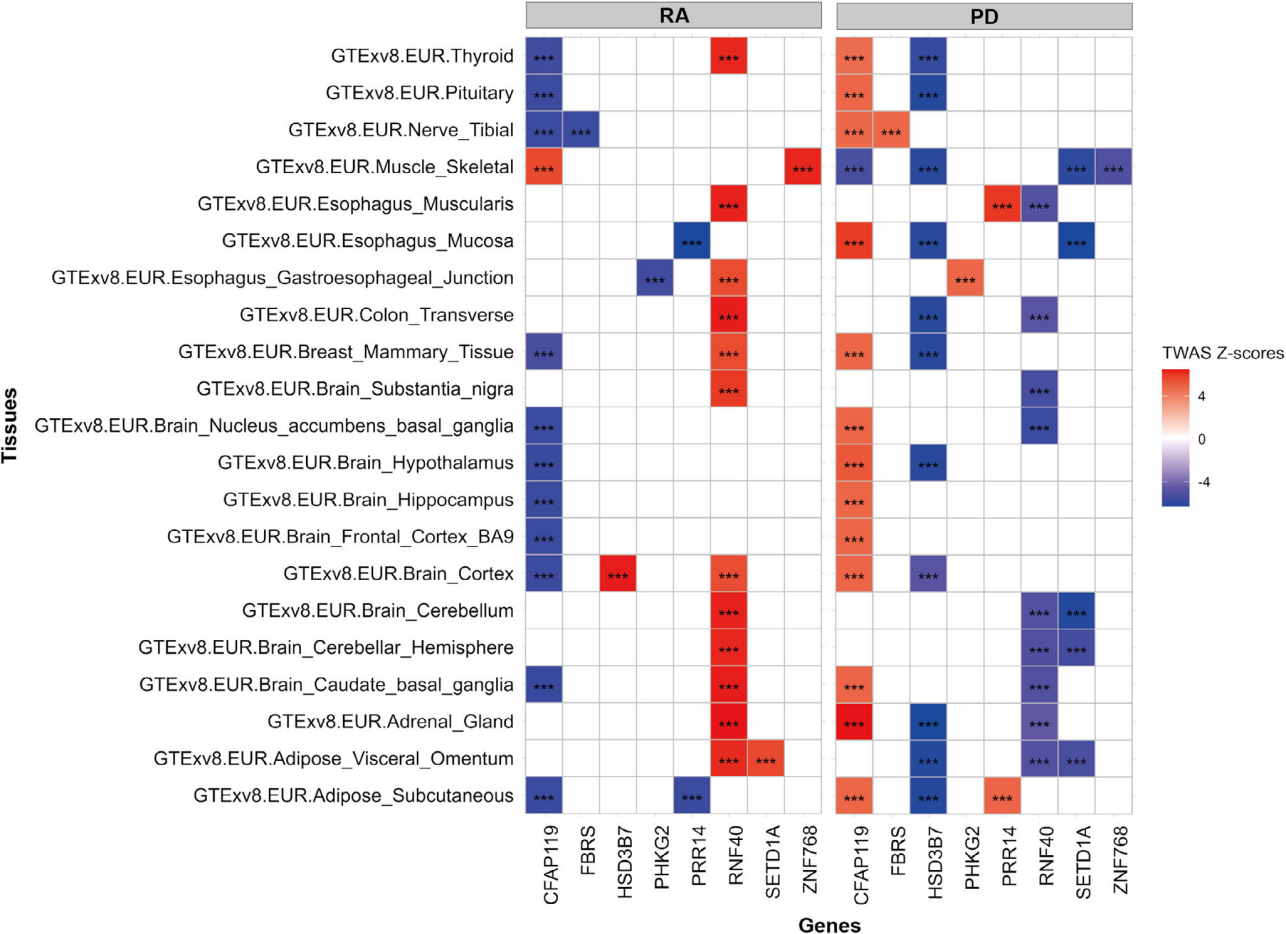
Heatmap of TWAS Z‐scores for selected genes associated with Rheumatoid arthritis (RA) and Parkinson's disease (PD) across GTEx tissues.

Finally, to confirm whether these TWAS signals reflected shared causal variants rather than correlated associations, we performed Bayesian colocalization analysis (COLOC) using cis‐eQTL summary statistics (PP4> 0.95). We identified 143 colocalized gene–tissue pairs for RA and 22 for PD (Table S9 and S10). Notably, *HSD3B7* exhibited strong colocalization signals for both diseases within the same tissue context, suggesting shared pleiotropy (Table S11). Together, these results delineate a spectrum of shared genetic signals between RA and PD, with *RNF40* emerging as a robust, cross‐tissue, directionally consistent candidate for mechanistic investigation.

### 2.5 CIA‐Associated Arthritis Attenuates MPTP‐Induced Dopaminergic Injury in a Cross‐Disease Mouse Model

To experimentally validate the MR‐inferred protective effect of RA on PD and interrogate *RNF40* as the lead comorbidity gene, we asked whether collagen‐induced arthritis (CIA) modifies Parkinsonian neurodegeneration in a murine RA–PD comorbidity model. Before *Rnf40* perturbation, we first examined whether CIA altered endogenous RNF40 expression across disease states. Immunofluorescence staining and western blot analysis showed that RNF40 expression in joint tissues was increased in CIA‐NC and CIA+MPTP‐NC mice compared with sham‐NC and MPTP‐NC mice, whereas no obvious difference was observed between sham‐NC and MPTP‐NC mice or between CIA‐NC and CIA+MPTP‐NC mice (Figure S6A–D). By contrast, RNF40 expression in the midbrain remained comparable among sham‐NC, MPTP‐NC, CIA‐NC, and CIA+MPTP‐NC mice (Figure S6E–H). These findings indicate that CIA‐associated arthritis induces endogenous RNF40 expression in joint tissues, whereas midbrain RNF40 levels are not overtly altered by CIA, MPTP, or their combination.

Efficient systemic AAV‐mediated *Rnf40* knockdown (*Rnf40*‐KD) was confirmed in joint and midbrain tissues (Figure 6A, Figure S7A). All in vivo procedures followed the prespecified protocol and timeline (Figure 1). CIA‐NC mice (CIA + control AAV) developed robust arthritis, including increased paw swelling, heightened mechanical hypersensitivity, impaired rotarod performance, and higher clinical arthritis scores compared with sham‐NC mice, confirming successful CIA induction (Figure 6B–E). CIA+MPTP‐NC mice showed comparable joint inflammation and arthritis severity to CIA‐NC mice, indicating that MPTP‐induced neurodegeneration did not appreciably modify the arthritis‐related phenotype. Consistently, micro‐CT and histology of knee and ankle joints revealed marked bone erosion and synovitis in both CIA‐NC and CIA+MPTP‐NC groups, with no meaningful between‐group differences (Figure 6F–K). Together, these joint readouts argue against a substantial causal impact of MPTP‐induced dopaminergic injury on the severity of arthritis‐related phenotypes in this model.

**FIGURE 6:**
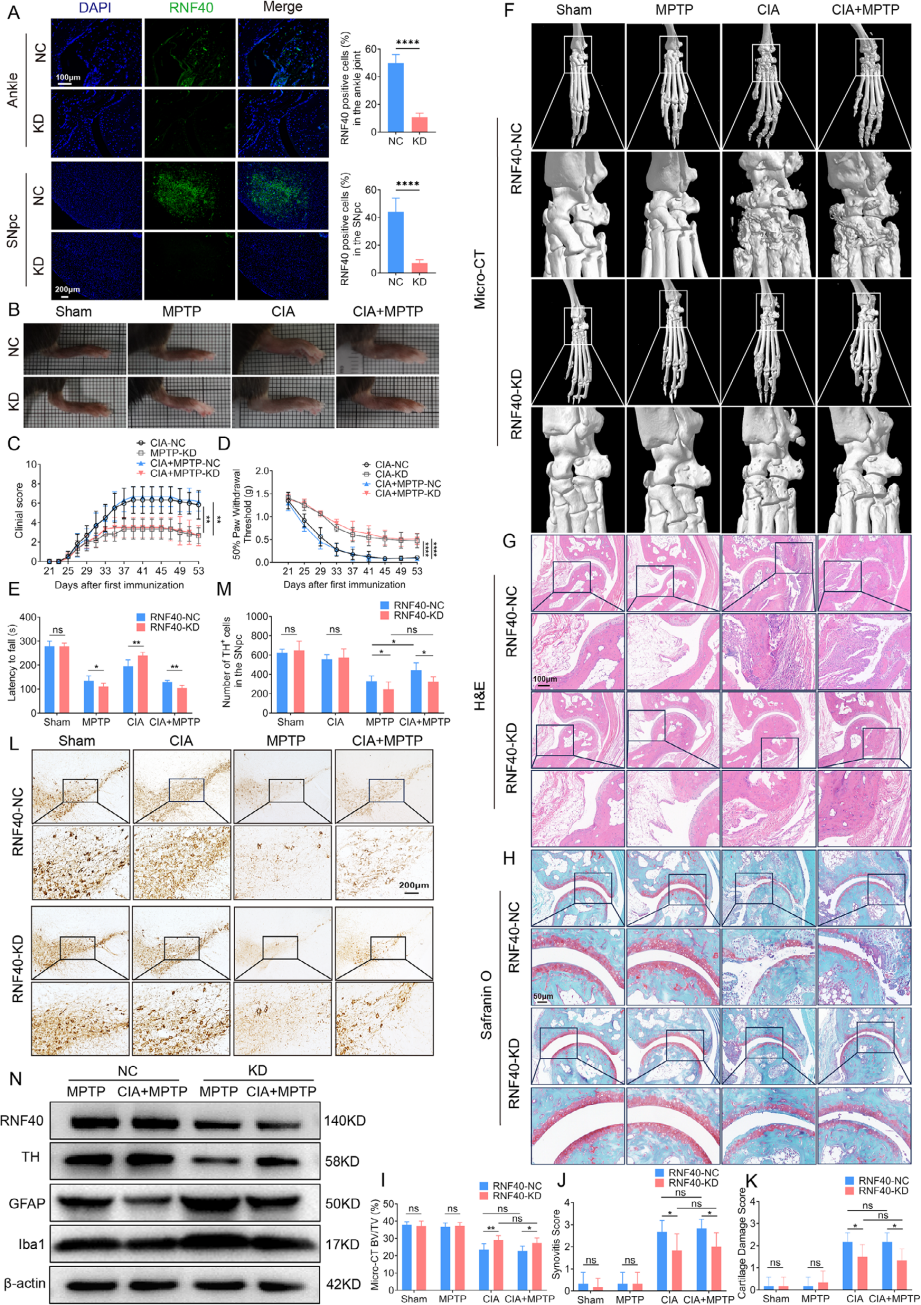
*Rnf40* functionally modulates CIA‐associated arthritis and MPTP‐induced dopaminergic injury in a cross‐disease mouse model. (A) Representative immunofluorescence images and quantitative analysis of the percentage of RNF40‐positive cells (green) in the ankle joints and substantia nigra pars compacta (SNpc) following systemic administration (tail vein injection) of adeno‐associated virus (AAV) carrying *Rnf40*‐knockdown (*Rnf40*‐KD) or negative control (*Rnf40*‐NC) sequences (n = 6/group). Nuclei were counterstained with DAPI (blue). (B) Representative macroscopic images of hindpaw swelling at the experimental endpoint across different groups. (C) Clinical arthritis scores of the four limbs evaluated every other day starting from day 21 post‐primary immunization (n = 6/group). (D) The 50% paw withdrawal threshold assessed via the von Frey filament test every other day from day 21 post‐immunization (n = 6/group). (E) Rotarod test results demonstrating the latency to fall at the experimental endpoint (n = 6/group). (F) Representative three‐dimensional micro‐CT reconstructed images of the mouse hind ankle joints. (G, H) Representative images of H&E staining (G) and Safranin O staining (H) of the ankle joint sections. (I‐K) Quantitative analysis of bone volume/tissue volume (BV/TV) based on micro‐CT (I), alongside synovitis scores (J) and cartilage damage scores (K) evaluated from histological sections (n = 6/group). (L, M) Representative immunohistochemistry images (L) and quantification (M) of tyrosine hydroxylase (TH)‐positive dopaminergic neurons in the SNpc (n = 6/group). (N) Western blot analysis of RNF40, TH, GFAP, and Iba1 protein expression levels in the SNpc. Data are presented as the mean ± SD. Statistical significance was determined by unpaired Student's t‐test (A), two‐way repeated measures ANOVA followed by Sidak's multiple comparisons test (C, D), and two‐way ANOVA followed by Tukey's multiple comparisons test (E, I‐K, M). For two‐way ANOVA, disease condition and AAV treatment were used as the two factors. ^*^P<0.05, ^**^P<0.01, ^***^P<0.001, ****P<0.0001; ns, not significant.

In contrast, arthritis modified MPTP‐induced neurodegeneration outcomes. MPTP‐NC mice exhibited pronounced loss of TH+ dopaminergic neurons in the substantia nigra, accompanied by microglial and astrocytic activation and motor impairment. Strikingly, CIA+MPTP‐NC mice displayed attenuated dopaminergic neuron loss and reduced glial activation compared with MPTP‐NC controls (Figure 6L–N, Figure S7B, C). Thus, in this experimental setting, CIA‐associated arthritis attenuated MPTP‐induced dopaminergic injury, providing in vivo support for an interaction between peripheral inflammatory arthritis and Parkinsonian vulnerability.

### 2.6 Systemic *Rnf40* Knockdown Alleviates Arthritis‐Like Pathology but Worsens MPTP‐Induced Neurodegeneration and Weakens Arthritis‐Associated Neuroprotection

To dissect its mechanistic contribution, we evaluated *Rnf40*‐KD mice across disease states. Systemic *Rnf40* knockdown attenuated arthritis severity, resulting in reduced paw swelling, lower clinical scores, reduced mechanical hypersensitivity, improved mobility, decreased synovial hyperplasia, and diminished bone erosion (Figure 6B–K). Given the systemic route of AAV‐shRNA delivery, this effect may reflect combined actions on joint tissues and peripheral immune compartments rather than a joint‐restricted mechanism. These data suggest that *Rnf40* contributes to CIA‐driven inflammatory pathology.

In stark contrast, *Rnf40*‐KD exacerbated PD‐related outcomes, driving greater TH+ neuron loss, heightened glial activation, and worsened motor deficits (Figure 6E, L–N, Figure S7B, C), suggesting that systemic *Rnf40* perturbation increases vulnerability to MPTP‐induced dopaminergic injury. Critically, *Rnf40* knockdown also eroded the arthritis‐associated neuroprotection: CIA+MPTP mice lacking *Rnf40* no longer exhibited the attenuated dopaminergic loss observed in CIA+MPTP‐NC animals, and their motor function was also impaired (Figure 6E, L–N, Figure S7B, C).

Taken together, these experiments nominate RNF40 as a candidate context‐dependent molecular link in the RA–PD axis, whereby systemic *Rnf40* perturbation alleviates inflammatory joint pathology but exacerbates MPTP‐induced dopaminergic neurodegeneration and weakens the arthritis‐associated neuroprotective phenotype suggested by human genetic analyses.

## 3 Discussion

To our knowledge, this is the first study to systematically triangulate population level associations, genetic causal inference, transcriptomic profiling, and in vivo functional validation to map the arthritis‐neurodegeneration axis within a single coherent framework. This stepwise design moves beyond correlational descriptions, yielding a directional, testable model linking joint immune pathology to central neurodegenerative vulnerability through tractable molecular nodes.

Using Cox proportional hazards models in UK Biobank, we found that OA was prospectively associated with increased risks of AD, DANS, and, more modestly, PD, whereas RA was associated with increased AD risk but not with incident PD. By accounting for follow‐up time and censoring, these time‐to‐event analyses better align the observational component with the prospective design of UK Biobank. Importantly, the Cox results do not provide independent observational evidence for a protective RA–PD association. Thus, the UK Biobank findings should be interpreted as population‐level evidence linking arthritis to selected neurodegenerative outcomes, rather than as direct causal evidence for the RA–PD relationship. In this framework, the RA–PD axis is mainly supported by the genetic, transcriptomic, and experimental layers, underscoring the value of multi‐layer triangulation when studying immune–neural crosstalk. Restricted medication‐related sensitivity analyses further supported this interpretation, while residual phenotype misclassification and treatment‐related confounding cannot be fully excluded in an observational biobank setting.

Our OA findings align with prior cohort evidence linking OA to AD, PD and DANS [15, 25, 26], and experimental work increasingly supports inflammatory coupling between joint pathology and neurodegenerative processes [1, 27]. Although these findings do not establish causality for OA, they support incorporating OA into a cross‐system risk framework in which cognitive, motor and autonomic outcomes merit parallel attention [28]. For RA, variability in reported RA–AD and RA–PD associations across regions likely reflects differences in therapeutic eras [14, 29, 30, 31], access to immunomodulatory therapy and background risk structures [32, 33, 34]. Therefore, neurodegenerative risk assessment in RA should be stratified by patient modifiers and treatment context rather than summarized as a single uniform effect.

Mechanistically, our genetic and transcriptomic analyses converged on a protective causal link from RA to PD, corroborated by our in vivo model. Transcriptome‐wide analyses revealed divergent transcriptional profiles between RA and PD across multiple tissues, consistent with opposing regulation of inflammatory programs and neurodegenerative vulnerability [35]. Integrating GWAS and TWAS, we prioritized *RNF40* based on cross‐tissue robustness and biological interpretability. *Rnf40* silencing attenuated arthritis yet exacerbated parkinsonian neuropathology and blunted the arthritis‐associated neuroprotective phenotype, supporting RNF40 as a tissue‐context‐dependent functional node rather than a passive correlate. However, the present data do not fully resolve the downstream pathways or cellular effectors through which RNF40 acts. RNF40 may influence the RA–PD phenotype through peripheral inflammatory remodeling, cytokine signaling, glial activation thresholds, blood–brain barrier regulation, neuron‐intrinsic stress responses, or interactions among these processes [36]. The CIA–MPTP paradigm should be viewed as a controlled functional model rather than a direct surrogate for natural human RA–PD comorbidity. It allows experimental testing of whether arthritis‐associated peripheral inflammation modifies dopaminergic vulnerability under neurotoxic stress, but it does not capture the chronic immune remodeling of RA, aging‐related susceptibility, α‐synuclein pathology, or the progressive course of idiopathic PD [37]. Accordingly, we interpret the animal data as functional evidence for cross‐system immune–neural interaction and *Rnf40* involvement, rather than as a full experimental reconstruction of the human comorbidity process.

Clinically, these results support a transition from single disease management toward cross‐system risk stratification. For OA, they argue for heightened vigilance for long‐term cognitive and autonomic outcomes alongside early metabolic and lifestyle optimization [38]. For RA, risk interpretation for AD, PD, and MS must incorporate age, metabolic status, and treatment history. Prospective studies are needed to determine whether sustained immunomodulation causally reshapes neurodegenerative trajectories [39]. RNF40 provides a tangible translational handle, most plausibly as a stratification biomarker and pathway node for cross‐system intervention rather than a replacement for established therapies.

Several limitations warrant consideration. First, composite endpoints may introduce non‐differential misclassification, attenuating effects; replication with refined clinical phenotypes, imaging measures and biomarker stratification is needed. Second, analyses were conducted predominantly in European ancestry UK Biobank participants; multi‐ancestry validation is required to assess generalizability. Third, the absence of summary level GWAS data for DANS and SMA precluded causal triangulation for these outcomes, so these signals should be interpreted as associations. Fourth, although *RNF40* was prioritized for robustness and plausibility, additional TWAS‐implicated genes may act through parallel networks. Notably, although *HSD3B7* showed robust RA–PD colocalization, its biological role and causal direction require in vivo validation. Fifth, the in vivo experiments were performed exclusively in male mice, which may limit the generalizability of the animal findings. Considering the known sex differences in RA, PD, and immune‐neuroinflammatory responses, future sex‐stratified studies are needed to determine whether the RNF40‐mediated joint–brain axis is conserved in females. Sixth, because AAV‐shRNA was delivered systemically, the present study cannot fully distinguish the tissue‐ or cell‐type‐specific contributions of *Rnf40*. Thus, these findings support the overall functional involvement of RNF40 in the RA–PD framework, while the relative contributions of peripheral immune cells, joint‐resident cells, and CNS compartments remain to be defined. Seventh, the behavioral assessment was limited to rotarod testing and mechanical sensitivity, which were selected to capture motor coordination and arthritis‐related mechanical hypersensitivity in the RA–PD comorbidity model. Future studies incorporating open‐field testing, gait analysis, and complementary motor assays will help define subtler Parkinsonian behavioral changes. These limitations may affect effect‐size precision and generalizability, but they do not negate the broader cross‐layer evidence linking arthritis‐related immune states to neurodegenerative vulnerability. Overall, the findings support a genetically informed and experimentally testable RA–PD axis, with RNF40 emerging as a context‐dependent joint–brain candidate node.

## 4 Experimental Section

### 4.1 Study Design and Reporting

We implemented a three‐tier design: (i) population‐based observational analyses in the UK Biobank (UKB), (ii) genetic inference using Bidirectional Mendelian Randomization (BMR) and transcriptome‐wide association study (TWAS), and (iii) in vivo validation of prioritized comorbidity genes. Reporting followed STROBE (observational), STROBE‐MR (MR) and ARRIVE (animal studies).

### 4.2 Data Sources and Study Population

We analyzed UKB, a prospective cohort of ∼500,000 individuals aged 40–69 years recruited across the UK from 2006 onward under Application Number 144904. Analyses were restricted to participants of European genetic ancestry. We applied UKB‐recommended genetic quality control, excluding genotyping outliers (Field 22010), individuals without principal components for genome‐wide analyses (Field 22020), individuals with excess relatedness (Field 22021), and participants with sex‐chromosome aneuploidy (Field 22019). To reduce reverse causation, participants with any target neurodegenerative diseases (NDDs) recorded at or before baseline were excluded. After exclusions, 310,162 participants remained. Public eQTL reference panels and GWAS summary statistics were used for BMR and TWAS (Table S1).

### 4.3 Exposures, Outcomes, and Covariates

Exposures (arthritis): Osteoarthritis (OA; M15‐M19) and rheumatoid arthritis (RA; M05‐M06) were defined using ICD‐10 diagnostic codes from linked hospital inpatient records and primary care records, including diagnoses recorded at baseline and during follow‐up. External GWAS summary statistics were used for OA (GCST007093) and RA (GCST90132223) (Table S12).

### 4.4 Outcomes (Neurodegenerative Diseases)

We evaluated five NDD outcomes defined by ICD‐10 codes: spinal muscular atrophy and related syndromes (SMA; G12), Parkinson's disease (PD; G20), Alzheimer's disease (AD; G30), multiple sclerosis (MS; G35), and disorders of the autonomic nervous system (DANS; G90). Publicly available GWAS summary statistics were used for AD (GCST90027158), PD (ieu‐b‐7) and MS (ieu‐b‐18) (Table S12). Summary‐level GWAS were unavailable for SMA and DANS.

### 4.5 Covariates (Observational Analyses)

Baseline covariates included age, sex, Townsend deprivation index, education level, body mass index (BMI), smoking status, alcohol use, history of cancer, coronary heart disease (CHD), type 2 diabetes (T2D), fracture history, and medication use (DMARDs, biologics/JAK inhibitors, glucocorticoids, or nonsteroidal anti‐inflammatory drugs).

### 4.6 Observational Statistical Analysis (UK Biobank)

Associations of OA and RA with incident NDDs were evaluated using Cox proportional hazards models, with hazard ratios (HRs) and 95% confidence intervals (CIs) reported. For each outcome‐specific analysis, participants with the corresponding NDD diagnosed at or before baseline were excluded. Follow‐up began at the baseline assessment date and continued until the first diagnosis of the corresponding NDD, death, withdrawal from UK Biobank, or the last available follow‐up date, whichever occurred first. OA and RA were treated as time‐varying exposures. Individuals with a diagnosis before or at baseline were considered exposed at baseline, and incident cases identified during follow‐up were updated as exposed during follow‐up.

We fitted two adjusted models, as follows: Model 1 was adjusted for age and sex; Model 2 was additionally adjusted for Townsend deprivation index, education level, BMI, smoking status, alcohol use, history of cancer, CHD, T2D, fracture history and medication use. We checked the proportional hazards assumption using Schoenfeld residuals after fitting the Cox proportional hazards model, and no evidence of violation was detected. We conducted prespecified subgroup analyses by sex, age group, BMI group, smoking status, and T2D status. To evaluate the robustness of our findings, we further performed a medication‐restricted sensitivity analysis by excluding participants with recorded use of DMARDs, biologics/JAK inhibitors, glucocorticoids, or NSAIDs. Logistic regression models were additionally used in supplementary analyses to evaluate the consistency between odds ratio estimates based on binary outcomes and hazard ratio estimates based on time‐to‐event outcomes.

We used two‐sided P<0.05 as the nominal threshold and controlled the false discovery rate (FDR) across multiple outcomes using the Benjamini–Hochberg procedure in secondary analyses. We performed all analyses in R (v3.5.3).

### 4.7 Bidirectional Mendelian Randomization

Instrument selection: For each MR analysis, we derived instruments from the exposure GWAS (OA, RA, AD, PD, or MS). We selected independent variants via PLINK clumping at genome‐wide significance (P<5×10^−^^8^; r^2^<0.001; 10,000 kb). We implemented bidirectional MR by alternately specifying arthritis traits and NDDs as exposures and outcomes.

### 4.8 Estimators and Sensitivity Analyses

We estimated causal effects using inverse‐variance weighted (IVW) MR with a random‐effects model. We probed robustness using MR‐Egger regression, weighted median, and weighted mode estimators. We assessed heterogeneity with Cochran's Q (IVW and MR‐Egger) and evaluated horizontal pleiotropy using the MR‐Egger intercept and the MR‐PRESSO global test; when MR‐PRESSO detected outliers, we reported outlier‐corrected IVW estimates. We performed leave‐one‐out analyses to identify influential instruments.

We implemented MR using the TwoSampleMR and MRPRESSO R packages and interpreted results against the three core assumptions: relevance, independence, and exclusion restriction.

### 4.9 Transcriptome‐Wide Association Study

We performed TWAS using the FUSION framework to test associations between genetically predicted gene expression and the traits analyzed in MR (OA, RA, AD, PD and MS) across multiple tissues. We used publicly available tissue‐specific eQTL weight panels (49 tissues) and European ancestry LD reference panels (http://gusevlab.org/projects/fusion/). Multiple testing was controlled using the Benjamini–Hochberg FDR procedure, and genes with FDR < 0.05 were considered statistically significant.

### 4.10 Colocalization Analysis

To evaluate whether GWAS and eQTL signals shared a common causal variant, we performed Bayesian colocalization analysis using COLOC. For trait–tissue pairs with significant TWAS signals, we extracted summary statistics for all SNPs within ±500 kb of the lead GWAS variant together with cis‐eQTL summary statistics from the corresponding tissue‐specific eQTL panels. We considered evidence for colocalization to be strong when the posterior probability for a shared causal variant (PP4) exceeded 0.95.

### 4.11 Patient and Public Involvement

Patients and/or the public were not involved in the design, or conduct, or reporting, or dissemination plans of this research.

### 4.12 Animal Studies, Housing, and Ethics

Male C57BL/6J mice (8 weeks old) were obtained from the Nanjing Medical University Laboratory Animal Center and maintained under specific pathogen–free (SPF) conditions (23–25°C; 45%–65% humidity; 12‐h light/dark cycle) with ad libitum access to standard chow (Research Diets D12450K) and water.

*Rnf40* knockdown (*Rnf40*‐KD) was achieved by systemic administration of an adeno‐associated virus (AAV) encoding shRNA targeting *Rnf*40 (pAAV‐U6‐shRNA (*Rnf40*)‐CMV‐Luc2‐WPRE); control mice received a matched empty‐vector AAV (pAAV‐U6‐shRNA (NC)‐CMV‐Luc2‐WPRE).

Mice were randomly assigned to control, MPTP, CIA or CIA+MPTP groups, each split into *Rnf40* knockdown and control subgroups (eight groups total). Sample sizes were prespecified a priori based on established Collagen‐induced arthritis (CIA) and subacute MPTP models, without post hoc power recalculation. All procedures were approved by the Institutional Animal Care and Use Committee of Nanjing Medical University (IACUC‐2511035) and conducted in accordance with ARRIVE guidelines. Randomization and blinded outcome assessment were applied where feasible.

### 4.13 Comorbidity Model

CIA was induced as described previously [40]. Chicken type II collagen (100 µg; Chondrex, 20011) emulsified 1:1 with complete Freund's adjuvant (Chondrex, 7023) was injected subcutaneously at the tail base on day 0, followed by a booster on day 21. Controls received saline. From day 21, arthritis severity was scored every 2 to 3 days (0 to 4 per limb; total 0 to 16).

To model subacute parkinsonian dopaminergic injury, mice received intraperitoneal MPTP (30 mg/kg; Sigma–Aldrich, M0896) once daily for five consecutive days. Control mice received an equivalent volume of saline.

CIA induction began on day 0 in the CIA and CIA+MPTP groups, whereas control and MPTP groups underwent sham procedures. MPTP administration commenced on day 42 in the MPTP and CIA+MPTP groups. AAV was delivered via tail‐vein injection on day −7 (100 µL; 1×10^13^ vg/ml). Behavioral testing was performed 7 days after the final MPTP injection, followed by euthanasia and tissue collection.

### 4.14 Behavioral Testing

Rotarod testMotor coordination was assessed using an accelerating rotarod (4–40 r.p.m. over 5 min). Latency to fall was averaged across three test trials after 3 days of training.

### 4.15 Mechanical Sensitivity

Mechanical allodynia was assessed using manual von Frey filaments (North Coast, USA) every 4 days from day 21 post‐induction until tissue collection. Following a 30‐min habituation on an elevated wire mesh, filaments were perpendicularly applied to the hind paw plantar surface. The 50% paw withdrawal threshold (PWT) was calculated using the up‐down method.

### 4.16 Micro‐CT

Fixed hind paws and tibiae were scanned using a Skyscan 1276 system (Bruker, Belgium) at 9 µm voxel resolution. Images were analyzed with CT‐Analyzer software. Bone volume fraction was quantified.

### 4.17 Histology

Decalcified ankle joints were paraffin‐embedded and stained with H&E and Safranin O. Synovitis was scored based on synovial lining hyperplasia and inflammatory infiltration.

### 4.18 Immunofluorescence

The joint sections were dewaxed, subjected to antigen retrieval, permeabilized, and blocked. Following fixation and washing, the brain sections underwent blocking. Sections were incubated with the primary antibodies listed in Table S13, followed by Alexa Fluor–conjugated secondary antibodies and DAPI counterstaining. Images were acquired by fluorescence microscopy (Leica, Germany), and signal intensity was quantified using ImageJ.

### 4.19 Immunohistochemistry

Brain sections were treated with 3% hydrogen peroxide and then blocked. Sections were incubated with the primary antibody, followed by streptavidin–HRP and DAB development. Images were acquired using an optical microscope (Olympus, Japan).

### 4.20 Western Blot

Total protein from the substantia nigra pars compacta (SNpc), striatum (STR) and ankle tissue was extracted and quantified via BCA assay. Equal protein aliquots (50 µg) were resolved by 12% SDS‐PAGE and transferred to PVDF membranes. After blocking, membranes were probed with primary antibodies overnight at 4°C, followed by incubation with HRP‐conjugated secondary antibodies. Immunoreactive bands were visualized via enhanced chemiluminescence (Amersham Imager 600, GE Healthcare, USA) and densitometrically analyzed using ImageJ software. RNF40, TH, and β‐actin were visualized on the same membrane using stripping and reprobing.

### 4.21 Statistical Analysis

Analyses were conducted using complete‐case data. Two‐sided statistical tests were applied where appropriate, model assumptions and robustness were assessed via prespecified sensitivity analyses. Coding lists and analysis scripts are available from the corresponding author upon reasonable request. In vivo data are presented as mean±SD. Graphs were generated using GraphPad Prism 10 software. Comparisons between two groups were performed using the unpaired Student's t‐test, whereas multiple comparisons were evaluated using one‐way or two‐way analysis of variance (ANOVA) followed by Tukey's or Sidak's multiple comparisons test, as appropriate. P values less than 0.05 were considered statistically significant.

## Author Contributions

JH, YL, PH, JW, and WX conceptualized the study, designed the experiments, and coordinated the research. WX curated and analyzed the bioinformatics data. JW, LY, ZW, and YH performed the in vivo animal experiments. JW and LY conducted the experimental sample evaluations and performed the statistical analyses. JW and WX drafted and critically revised the manuscript. JH, YL, and PH supervised the entire study and oversaw the project administration. All authors critically reviewed the manuscript for important intellectual content and approved the final version for submission. JH is responsible for the overall content as guarantor.

## Funding

This work was supported by the National Natural Science Foundation of China (82272544 and 81672218) and the Natural Science Foundation of Jiangsu Province (BK20251945).

## Ethics Approval Statement

All mouse experiments were approved by the Institutional Animal Care and Use Committee of Nanjing Medical University (IACUC‐2511035) and conducted in accordance with ARRIVE guidelines. The human‐related data analyzed in this study were extracted from publicly available databases containing de‐identified and completely anonymized data; therefore, the requirement for institutional ethical approval and patient informed consent was waived.

## Permission to Reproduce Material from Other Sources

Schematic elements in Figure 1 were created with BioRender under a publication license.

## Conflicts of Interest

The authors declare no conflicts of interest.

## Supporting information




**Supporting File**: advs76930‐sup‐0001‐SuppMat.docx.

## Data Availability

The individual‐level data from the UK Biobank analyzed in this study are available upon application to the UK Biobank (www.ukbiobank.ac.uk). The GWAS summary statistics and eQTL data used for Mendelian randomization and TWAS are publicly available and detailed in the Supplementary Materials. Data supporting the in vivo experimental findings are available from the corresponding author upon reasonable request.
