# Supplementary material for: Systematic Multi‐Level Analyses Decode the Arthritis‐Neurodegeneration Axis With In Vivo Validation
Source: Adv Sci (Weinh). 2026 Aug 5;13(60):e76930. doi: 10.1002/advs.76930 (PMC13440215; doi:10.1002/advs.76930)
Supplement: Supplementary file 1 [file ADVS-13-e76930-s001.docx]

Supporting Information

**Systematic Multi-Level Analyses Decode the Arthritis-Neurodegeneration Axis with In Vivo Validation**

**Authors:** *Jinwen Wang, Wenhui Xie, Lei Yang, Zhengrong Wang, Yi Huang, Peng Huang*, Yuan Liu*, Jun Hu**

**Supplemental Figures**

**Figure S1.** Forest plot of odds ratios for associations of OA/RA with neurodegenerative outcomes in logistic regression analyses.

**Figure S2.** Summary of MR analysis between PD and RA.

**Figure S3.** Summary of MR analysis between MS and RA.

**Figure S4.** Summary of MR analysis between RA and MS.

**Figure S5.** Summary of MR analysis between the remaining OA/RA and neurodegenerative diseases.

**Figure S6.** Endogenous RNF40 expression in joint and midbrain tissues across disease states.

**Figure S7.** Validation of AAV-mediated *Rnf40* knockdown and assessment of dopaminergic and neuroinflammatory markers in the mouse midbrain.

**Supplemental Tables**

**Table S1.** Baseline characteristics of the included participants in five neurodegenerative diseases in Cox proportional hazards analyses.

**Table S2.** Baseline characteristics of the included participants in five neurodegenerative diseases in Logistic regression analyses.

**Table S3.** Logistic regression analyses of OA/RA and incident neurodegenerative outcomes in UK Biobank.

**Table S4.** Sensitivity analyses regarding risk of incident five neurodegenerative diseases according to OA/RA.

**Table S5.** Summary for IVs in MR analysis.

**Table S6.** MR-PRESSO abnormal SNP report.

**Table S7.** Summary for RA TWAS results in tibial nerve.

**Table S8.** Summary for PD TWAS results in tibial nerve.

**Table S9.** Summary for pleiotropic genes of Parkinson's disease in 21 tissues.

**Table S10.** Summary for pleiotropic genes of Rheumatoid arthritis in 21 tissues.

**Table S11.** Coloc results of pleiotropic genes shared by RA and PD in 21 tissues.

**Table S12.** Descriptive characteristics of included GWAS datasets.

**Table S13.** Details of primary antibodies used in this study.

**Supplemental Figures**

**Figure S1.** Forest plot of odds ratios for associations of OA/RA with neurodegenerative outcomes in logistic regression analyses. (A) ORs associated with age for OA patients with neurodegenerative outcomes. (B) ORs associated with sex for OA patients with neurodegenerative outcomes. (C-E) ORs associated with BMI, smoking status and diabetes for OA patients with neurodegenerative outcomes. (F-J) ORs associated with age, sex, BMI, smoking status and diabetes for RA patients with neurodegenerative outcomes. All analyses were performed using multivariable logistic regression models.

**Figure S2.** Summary of MR analysis between PD and RA. (A) Forest plot of the causal effects of PD associated SNPs on RA. (B) Scatter plot showed causal effects of PD on RA. The slopes of line represent the causal effect of each method, respectively. (C) Funnel plot of the causal effect of PD on RA. (D) Leave-one-out analysis plots for PD on RA. MR, Mendelian randomization; SNP, single nucleotide polymorphism; RA, Rheumatoid arthritis; PD, Parkinson’s disease.

**Figure S3.** Summary of MR analysis between MS and RA. (A) Forest plot of the causal effects of MS associated SNPs on RA. (B) Scatter plot showed causal effects of MS on RA. The slopes of line represent the causal effect of each method, respectively. (C) Funnel plot of the causal effect of MS on RA. (D) Leave-one-out analysis plots for MS on RA. MR, Mendelian randomization; SNP, single nucleotide polymorphism; RA, Rheumatoid arthritis; MS, Multiple sclerosis.

**Figure S4.** Summary of MR analysis between RA and MS. (A) Forest plot of the causal effects of RA associated SNPs on MS. (B) Scatter plot showed causal effects of RA on MS. The slopes of line represent the causal effect of each method, respectively. (C) Funnel plot of the causal effect of RA on MS. (D) Leave-one-out analysis plots for RA on MS. MR, Mendelian randomization; SNP, single nucleotide polymorphism; RA, Rheumatoid arthritis; MS, Multiple sclerosis.

**Figure S5.** Summary of MR analysis between the remaining OA/RA and neurodegenerative diseases. (A) Forest plot of SNPs associated with OA and AD. (B) Forest plot of SNPs associated with AD and OA. (C) Forest plot of SNPs associated with OA and MS. (D) Forest plot of SNPs associated with MS and OA. (E) Forest plot of SNPs associated with OA and PD. (F) Forest plot of SNPs associated with PD and OA. (G) Forest plot of SNPs associated with RA and AD. (H) Forest plot of SNPs associated with AD and RA. MR, Mendelian randomization; SNP, single nucleotide polymorphism; OA, Osteoarthritis; RA, Rheumatoid arthritis; MS, Multiple sclerosis; AD, Alzheimer’s disease; PD, Parkinson’s disease.

**Figure S6.** Endogenous RNF40 expression in joint and midbrain tissues across disease states. (A, B) Representative immunofluorescence images and quantitative analysis of the percentage of RNF40-positive cells (green) in the ankle joints. (C, D) Western blot and quantitative analysis of RNF40 protein expression levels in the ankle joints. (E, F) Representative immunofluorescence images and quantitative analysis of the percentage of RNF40-positive cells (green) in the substantia nigra pars compacta (SNpc). (G, H) Western blot and quantitative analysis of RNF40 protein expression levels in the SNpc. Data are presented as the mean ± SD. Statistical significance was determined by one-way ANOVA followed by Tukey’s multiple comparisons test. *P<0.05, **P<0.01; ns, not significant.

**Figure S7.** Validation of AAV-mediated *Rnf40* knockdown and assessment of dopaminergic and neuroinflammatory markers in the mouse midbrain. (A) Representative in vivo bioluminescence images confirming successful brain and ankle transduction of the AAV-Luc vector. (B) Western blot analysis of RNF40, TH, GFAP, and Iba1 protein expression levels in the midbrain across Sham, CIA, MPTP, and CIA+MPTP groups. β-actin served as the internal loading control. Molecular weights are indicated in kilodaltons (kDa). (C, D) Representative immunofluorescence images depicting the activation of astrocytes and microglia in the midbrain, labeled with (C) GFAP (green) and (D) Iba1 (red), respectively. Nuclei were counterstained with DAPI (blue).

**Supplemental Tables**

| **Table S1.** Baseline characteristics of the included participants in five neurodegenerative diseases | | | | | | | | | | |
| --- | --- | --- | --- | --- | --- | --- | --- | --- | --- | --- |
|  | Participants, n (%) | | | | | | | | | |
| Characteristics | Overall  (n=310,162) | OA  (n=81,802) | NonOA  (n=228,360) | RA  (n=7,848) | NonRA  (n=302,314) | AD  (n=2,631) | PD  (n=2,240) | DANS  (n=363) | MS  (n=361) | SMA  (n=459) |
| OA |  |  |  |  |  |  |  |  |  |  |
| Yes | 81,802(26.4) | - | - | 4,658(59.4) | 77,144(25.5) | 1,151(43.7) | 941(42.0) | 140(38.6) | 103(28.5) | 138(30.1) |
| No | 228,360(73.6) | - | - | 3,190(40.6) | 225,170(74.5) | 1,480(56.3) | 1,299(58.0) | 223(61.4) | 258(71.5) | 321(69.9) |
| RA |  |  |  |  |  |  |  |  |  |  |
| Yes | 7,848(2.5) | 4,658(5.7) | 3,190(1.4) | - | - | 127(4.8) | 88(3.9) | 19(5.2) | 8(2.2) | 17(3.7) |
| No | 302,314(97.5) | 77,144(94.3) | 225,170(98.6) | - | - | 2,504(95.2) | 2,152(96.1) | 344(94.8) | 353(97.8) | 442(96.3) |
| Age, mean(SD) | 56.65±8.016 | 59.80±6.844 | 55.52±8.105 | 59.36±7.133 | 56.58±8.026 | 64.64±4.239 | 62.83±5.283 | 59.39±7.296 | 54.33±8.108 | 60.80±6.509 |
| Sex |  |  |  |  |  |  |  |  |  |  |
| Male | 143,618(46.3) | 33,517(41.0) | 110,101(48.2) | 2,679(34.1) | 140,939(46.6) | 1,277(48.5) | 1,424(63.6) | 202(55.6) | 110(30.5) | 264(57.5) |
| Female | 166,544(53.7) | 48,285(59.0) | 118,259(51.8) | 5,169(65.9) | 161,375(53.4) | 1,354(51.5) | 816(36.4) | 161(44.4) | 251(69.5) | 195(42.5) |
| TDI, mean(SD) | -1.47±3.000 | -1.37±3.039 | -1.51±2.986 | -1.01±3.227 | -1.48±2.994 | -1.22±3.173 | -1.54±2.986 | -0.98±3.444 | -1.22±3.062 | -1.45±3.101 |
| Education |  |  |  |  |  |  |  |  |  |  |
| College or University degree | 106,340(34.4) | 21,413(26.2) | 84,927(37.2) | 1,825(23.3) | 104,515(34.6) | 562(21.4) | 658(29.4) | 133(36.6) | 121(33.5) | 142(30.9) |
| A levels/AS levels or equivalent | 36,322(11.7) | 8,224(10.1) | 28,098(12.3) | 765(9.7) | 35,557(11.8) | 210(8.0) | 219(9.8) | 36(9.9) | 51(14.1) | 44(9.6) |
| O levels/GCSEs or equivalent | 65,049(21.0) | 17,790(21.7) | 47,259(20.7) | 1,657(21.1) | 63,392(21.0) | 508(19.3) | 446(19.9) | 66(18.2) | 73(20.2) | 99(21.6) |
| CSEs or equivalent | 15,456(5.0) | 3,675(4.5) | 11,781(5.2) | 357(4.5) | 15,099(5.0) | 51(1.9) | 71(3.2) | 6(1.7) | 24(6.6) | 18(3.9) |
| NVQ or HND or HNC or equivalent | 19,331(6.2) | 5,756(7.0) | 13,575(5.9) | 542(6.9) | 18,789(6.2) | 180(6.8) | 157(7.0) | 23(6.3) | 12(3.3) | 26(5.7) |
| Other professional qualifications | 15,888(5.1) | 5,076(6.2) | 10,812(4.7) | 489(6.2) | 15,399(5.1) | 158(6.0) | 127(5.7) | 17(4.7) | 19(5.3) | 24(5.2) |
| None of the above | 48,766(15.7) | 18,830(23.0) | 29,936(13.1) | 2,103(26.8) | 46,663(15.4) | 905(34.4) | 531(23.7) | 80(22.0) | 59(16.3) | 104(22.7) |
| Prefer not to answer | 2,698(0.9) | 938(1.1) | 1,760(0.8) | 104(1.3) | 2,594(0.9) | 54(2.1) | 26(1.2) | 2(0.6) | 2(0.6) | 2(0.4) |
| NA | 312(0.1) | 100(0.1) | 212(0.1) | 6(0.1) | 306(0.1) | 3(0.1) | 5(0.2) | 0(0.0) | 0(0.0) | 0(0.0) |
| BMI |  |  |  |  |  |  |  |  |  |  |
| ＜18.5 | 1,599(0.5) | 261(0.3) | 1,338(0.6) | 50(0.6) | 1,549(0.5) | 8(0.3) | 9(0.4) | 5(1.4) | 1(0.3) | 2(0.4) |
| ≥18.5 to ＜25.0 | 103,040(33.2) | 19,665(24.0) | 83,375(36.5) | 2,074(26.4) | 100,966(33.4) | 791(30.1) | 620(27.7) | 105(28.9) | 128(35.5) | 146(31.8) |
| ≥25.0 to ＜30.0 | 130,938(42.2) | 34,035(41.6) | 96,903(42.4) | 3,102(39.5) | 127,836(42.3) | 1,160(44.1) | 1,036(46.3) | 155(42.7) | 140(38.8) | 203(44.2) |
| ＞30.0 | 73,548(23.7) | 27,479(33.6) | 46,069(20.2) | 2,564(32.7) | 70,984(23.5) | 656(24.9) | 558(24.9) | 88(24.2) | 88(24.4) | 101(22.0) |
| NA | 1,037(0.3) | 362(0.4) | 675(0.3) | 58(0.7) | 979(0.3) | 16(0.6) | 17(0.8) | 10(2.8) | 4(1.1) | 7(1.5) |
| Smoking |  |  |  |  |  |  |  |  |  |  |
| Never | 167,254(53.9) | 40,777(49.9) | 126,477(55.4) | 3,551(45.2) | 163,703(54.1) | 1,242(47.2) | 1,141(50.9) | 180(49.6) | 170(47.1) | 228(49.7) |
| Previous | 109,678(35.4) | 32,579(39.8) | 77,099(33.8) | 3,264(41.6) | 106,414(35.2) | 1,140(43.3) | 933(41.7) | 136(37.5) | 115(31.9) | 183(39.9) |
| Current | 32,158(10.4) | 8,078(9.9) | 24,080(10.5) | 994(12.7) | 31,164(10.3) | 227(8.6) | 154(6.9) | 46(12.7) | 73(20.2) | 45(9.8) |
| Prefer not to answer | 1,064(0.3) | 365(0.4) | 699(0.3) | 39(0.5) | 1,025(0.3) | 22(0.8) | 12(0.5) | 1(0.3) | 3(0.8) | 3(0.7) |
| NA | 8(0.0) | 3(0.0) | 5(0.0) | 0(0.0) | 8(0.0) | 0(0.0) | 0(0.0) | 0(0.0) | 0(0.0) | 0(0.0) |
| Alcohol |  |  |  |  |  |  |  |  |  |  |
| Never | 9,667(3.1) | 3,132(3.8) | 6,535(2.9) | 425(5.4) | 9,242(3.1) | 159(6.0) | 98(4.4) | 20(5.5) | 7(1.9) | 23(5.0) |
| Previous | 10,710(3.5) | 3,551(4.3) | 7,159(3.1) | 521(6.6) | 10,189(3.4) | 145(5.5) | 124(5.5) | 25(6.9) | 25(6.9) | 17(3.7) |
| Current | 289,491(93.3) | 75,019(91.7) | 214,472(93.9) | 6,898(87.9) | 282,593(93.5) | 2,320(88.2) | 2,011(89.8) | 318(87.6) | 329(91.1) | 419(91.3) |
| Prefer not to answer | 286(0.1) | 97(0.1) | 189(0.1) | 4(0.1) | 282(0.1) | 7(0.3) | 7(0.3) | 0(0.0) | 0(0.0) | 0(0.0) |
| NA | 8(0.0) | 3(0.0) | 5(0.0) | 0(0.0) | 8(0.0) | 0(0.0) | 0(0.0) | 0(0.0) | 0(0.0) | 0(0.0) |
| Cancer |  |  |  |  |  |  |  |  |  |  |
| Yes | 24,235(7.8) | 7,615(9.3) | 16,620(7.3) | 699(8.9) | 23,536(7.8) | 276(10.5) | 192(8.6) | 32(8.8) | 30(8.3) | 40(8.7) |
| No | 285,000(91.9) | 73,884(90.3) | 211,116(92.4) | 7,110(90.6) | 277,890(91.9) | 2,345(89.1) | 2,044(91.3) | 328(90.4) | 328(90.9) | 417(90.8) |
| Do not know | 794(0.3) | 265(0.3) | 529(0.2) | 32(0.4) | 762(0.3) | 7(0.3) | 4(0.2) | 1(0.3) | 3(0.8) | 2(0.4) |
| Prefer not to answer | 123(0.0) | 35(0.0) | 88(0.0) | 7(0.1) | 116(0.0) | 3(0.1) | 0(0.0) | 2(0.6) | 0(0.0) | 0(0.0) |
| NA | 10(0.0) | 3(0.0) | 7(0.0) | 0(0.0) | 10(0.0) | 0(0.0) | 0(0.0) | 0(0.0) | 0(0.0) | 0(0.0) |
| CHD |  |  |  |  |  |  |  |  |  |  |
| Yes | 90,465(29.2) | 31,081(38.0) | 59,384(26.0) | 3,170(40.4) | 87,295(28.9) | 1,176(44.7) | 1,006(44.9) | 139(38.3) | 108(29.9) | 167(36.4) |
| No | 219,171(70.7) | 50,540(61.8) | 168,631(73.8) | 4,656(59.3) | 214,515(71.0) | 1,444(54.9) | 1,231(55.0) | 222(61.2) | 250(69.3) | 291(63.4) |
| Prefer not to answer | 517(0.2) | 178(0.2) | 339(0.1) | 22(0.3) | 495(0.2) | 11(0.4) | 3(0.1) | 2(0.6) | 3(0.8) | 1(0.2) |
| NA | 9(0.0) | 3(0.0) | 6(0.0) | 0(0.0) | 9(0.0) | 0(0.0) | 0(0.0) | 0(0.0) | 0(0.0) | 0(0.0) |
| Diabetes |  |  |  |  |  |  |  |  |  |  |
| Yes | 14,861(4.8) | 5,404(6.6) | 9,457(4.1) | 607(7.7) | 14,254(4.7) | 296(11.3) | 213(9.5) | 41(11.3) | 23(6.4) | 29(6.3) |
| No | 295,301(95.2) | 76,398(93.4) | 218,903(95.9) | 7,241(92.3) | 288,060(95.3) | 2,335(88.7) | 2,027(90.5) | 322(88.7) | 338(93.6) | 430(93.7) |
| Fracture History |  |  |  |  |  |  |  |  |  |  |
| Yes | 29,753(9.6) | 9,350(11.4) | 20,403(8.9) | 967(12.3) | 28,786(9.5) | 286(10.9) | 203(9.1) | 53(14.6) | 42(11.6) | 41(8.9) |
| No | 280,409(90.4) | 72,452(88.6) | 207,957(91.1) | 6,881(87.7) | 273,528(90.5) | 2,345(89.1) | 2,037(90.9) | 310(85.4) | 319(88.4) | 418(91.1) |
| Medication History |  |  |  |  |  |  |  |  |  |  |
| Yes | 82,800(26.7) | 27,293(33.4) | 55,507(24.3) | 3,908(49.8) | 78,892(26.1) | 915(34.8) | 739(33.0) | 113(31.1) | 110(30.5) | 131(28.5) |
| No | 227,362(73.3) | 54,509(66.6) | 172,853(75.7) | 3,940(50.2) | 223,422(73.9) | 1,716(65.2) | 1,501(67.0) | 250(68.9) | 251(69.5) | 328(71.5) |
| Abbreviations: SMA, Spinal muscular atrophy and related syndromes; PD, Parkinson’s disease; AD, Alzheimer’s disease; MS, Multiple sclerosis; DANS, Disorders of autonomic nervous system; OA, Osteoarthritis; RA, Rheumatoid Arthritis; SD, standard deviation; BMI, body mass index; CHD, coronary heart disease; TDI, Townsend deprivation index. | | | | | | | | | | |

| **Table S2.** Baseline characteristics of the included participants in five neurodegenerative diseases in Logistic regression analyses | | | | | | | | | | |
| --- | --- | --- | --- | --- | --- | --- | --- | --- | --- | --- |
|  | Participants, n (%) | | | | | | | | | |
| Characteristics | Overall  (n=310,162) | OA  (n=81,802) | NonOA  (n=228,360) | RA  (n=7,848) | NonRA  (n=302,314) | AD  (n=2,625) | PD  (n=2,784) | DANS  (n=520) | MS  (n=1,589) | SMA  (n=520) |
| OA |  |  |  |  |  |  |  |  |  |  |
| Yes | 81,802(26.4) | - | - | 4,658(59.4) | 77,144(25.5) | 1,144(43.6) | 1,134(40.7) | 196(37.7) | 403(25.4) | 153(29.4) |
| No | 228,360(73.6) | - | - | 3,190(40.6) | 225,170(74.5) | 1,481(56.4) | 1,650(59.3) | 324(62.3) | 1,186(74.6) | 367(70.6) |
| RA |  |  |  |  |  |  |  |  |  |  |
| Yes | 7,848(2.5) | 4,658(5.7) | 3,190(1.4) | - | - | 108(4.1) | 65(2.3) | 18(3.5) | 8(0.5) | 14(2.7) |
| No | 302,314(97.5) | 77,144(94.3) | 225,170(98.6) | - | - | 2,517(95.9) | 2,719(97.7) | 502(96.5) | 1,581(99.5) | 506(97.3) |
| Age, mean(SD) | 56.65±8.016 | 59.80±6.844 | 55.52±8.105 | 59.36±7.13 | 56.58±8.03 | 64.64±4.232 | 62.68±5.376 | 58.95±7.553 | 55.34±7.620 | 60.47±6.694 |
| Sex |  |  |  |  |  |  |  |  |  |  |
| Male | 143,618(46.3) | 33,517(41.0) | 110,101(48.2) | 2,679(34.1) | 140,939(46.6) | 1,277(48.6) | 1,788(64.2) | 289(55.6) | 443(27.9) | 302(58.1) |
| Female | 166,544(53.7) | 48,285(59.0) | 118,259(51.8) | 5,169(65.9) | 161,375(53.4) | 1,348(51.4) | 996(35.8) | 231(44.4) | 1,146(72.1) | 218(41.9) |
| TDI, mean(SD) | -1.47±3.00 | -1.37±3.04 | -1.51±2.99 | -1.01±3.23 | -1.48±2.99 | -1.22±3.18 | -1.56±2.97 | -0.97±3.43 | -1.48±3.06 | -1.40±3.09 |
| Education |  |  |  |  |  |  |  |  |  |  |
| College or University degree | 106,340(34.3) | 21,413(26.2) | 84,927(37.2) | 1,825(23.3) | 104,515(34.6) | 563(21.5) | 834(30.0) | 178(34.2) | 571(36.0) | 162(31.2) |
| A levels/AS levels or equivalent | 36,322(11.7) | 8,224(10.1) | 28,098(12.3) | 765(9.8) | 35,557(11.8) | 211(8.0) | 280(10.1) | 61(11.7) | 221(13.9) | 50(9.6) |
| O levels/GCSEs or equivalent | 65,049(21.0) | 17,790(21.8) | 47,259(20.7) | 1,657(21.1) | 63,392(21.0) | 509(19.4) | 544(19.6) | 100(19.2) | 346(21.8) | 111(21.3) |
| CSEs or equivalent | 15,456(5.0) | 3,675(4.5) | 11,781(5.2) | 357(4.6) | 15,099(5.0) | 51(1.9) | 83(3.0) | 12(2.3) | 82(5.2) | 23(4.4) |
| NVQ or HND or HNC or equivalent | 19,331(6.2) | 5,756(7.0) | 13,575(6.0) | 542(6.9) | 18,789(6.2) | 180(6.9) | 198(7.1) | 29(5.6) | 59(3.7) | 32(6.2) |
| Other professional qualifications | 15,888(5.1) | 5,076(6.2) | 10,812(4.7) | 489(6.2) | 15,399(5.1) | 158(6.0) | 161(5.8) | 27(5.2) | 77(4.8) | 26(5.0) |
| None of the above | 48,766(15.7) | 18,830(23.0) | 29,936(13.1) | 2,103(26.8) | 46,663(15.5) | 897(34.2) | 644(23.2) | 108(20.8) | 219(13.8) | 114(21.9) |
| Prefer not to answer | 2,698(0.9) | 938(1.1) | 1,760(0.8) | 104(1.3) | 2,594(0.9) | 53(2.0) | 33(1.2) | 5(1.0) | 13(0.8) | 2(0.4) |
| NA | 312(0.1) |  |  |  |  |  |  |  |  |  |
| BMI |  |  |  |  |  |  |  |  |  |  |
| ＜18.5 | 1,587(0.5) | 259(0.3) | 1,328(0.6) | 50(0.6) | 1,537(0.5) | 8(0.3) | 14(0.5) | 5(1.0) | 16(1.1) | 3(0.6) |
| ≥18.5 to ＜25.0 | 102,773(33.1) | 19,614(24.1) | 83,159(36.5) | 2,069(26.6) | 100,704(33.4) | 787(30.2) | 777(28.2) | 156(30.9) | 587(40.0) | 164(32.5) |
| ≥25.0 to ＜30.0 | 130,971(42.2) | 34,027(41.8) | 96,944(42.6) | 3,101(39.8) | 127,870(42.4) | 1,155(44.3) | 1,283(46.6) | 215(42.6) | 563(37.7) | 223(44.2) |
| ＞30.0 | 73,687(23.8) | 27,519(33.8) | 46,168(20.3) | 2,567(33.0) | 71,120(23.6) | 656(25.2) | 682(24.7) | 129(25.5) | 316(21.2) | 115(22.8) |
| NA | 1,144(0.4) |  |  |  |  |  |  |  |  |  |
| Smoking |  |  |  |  |  |  |  |  |  |  |
| Never | 167,254(53.9) | 40,777(49.9) | 126,477(55.4) | 3,551(45.2) | 163,703(54.2) | 1,236(47.1) | 1,469(52.8) | 248(47.7) | 741(46.6) | 254(48.8) |
| Previous | 109,678(35.4) | 32,579(39.8) | 77,099(33.8) | 3,264(41.6) | 106,414(35.2) | 1,140(43.4) | 1,112(39.9) | 203(39.0) | 584(36.8) | 211(40.6) |
| Current | 32,158(10.4) | 8,078(9.9) | 24,080(10.5) | 994(12.7) | 31,164(10.3) | 227(8.6) | 186(6.7) | 68(13.1) | 254(16.0) | 51(9.8) |
| Prefer not to answer | 1,064(0.3) | 365(0.4) | 699(0.3) | 39(0.5) | 1,025(0.3) | 22(0.8) | 17(0.6) | 1(0.2) | 10(0.6) | 4(0.8) |
| NA | 8(0.3) |  |  |  |  |  |  |  |  |  |
| Alcohol |  |  |  |  |  |  |  |  |  |  |
| Never | 9,667(3.1) | 3,132(3.8) | 6,535(2.9) | 425(5.4) | 9,242(3.1) | 161(6.1) | 132(4.7) | 27(5.2) | 57(3.6) | 24(4.6) |
| Previous | 10,710(3.5) | 3,551(4.3) | 7,159(3.1) | 521(6.6) | 10,189(3.4) | 146(5.6) | 175(6.3) | 34(6.5) | 102(6.4) | 22(4.2) |
| Current | 289,491(93.3) | 75,019(91.7) | 214,472(93.9) | 6,898(87.9) | 282,593(93.5) | 2,311(88.0) | 2,470(88.7) | 459(88.3) | 1,430(90.0) | 474(91.2) |
| Prefer not to answer | 286(1.1) | 97(0.1) | 189(0.1) | 4(0.1) | 282(0.1) | 7(0.3) | 7(0.3) | 0(0.0) | 0(0.0) | 0(0.0) |
| NA | 8(0.0) |  |  |  |  |  |  |  |  |  |
| Cancer |  |  |  |  |  |  |  |  |  |  |
| Yes | 24,235(7.8) | 7,615(9.3) | 16,620(7.3) | 699(8.9) | 23,536(7.8) | 276(10.5) | 254(9.1) | 52(10.0) | 125(7.9) | 47(9.0) |
| No | 285,000(91.9) | 73,884(90.3) | 211,116(92.5) | 7,110(90.6) | 277,890(91.9) | 2,339(89.1) | 2,527(90.8) | 465(89.4) | 1,455(91.6) | 471(90.6) |
| Do not know | 794(0.3) | 265(0.3) | 529(0.2) | 32(0.4) | 762(0.3) | 7(0.3) | 3(0.1) | 1(0.2) | 9(0.6) | 2(0.4) |
| Prefer not to answer | 123(0.0) | 35(0.0) | 88(0.0) | 7(0.1) | 116(0.0) | 3(0.1) | 0(0.0) | 2(0.4) | 0(0.0) | 0(0.0) |
| NA | 10(0.0) |  |  |  |  |  |  |  |  |  |
| CHD |  |  |  |  |  |  |  |  |  |  |
| Yes | 90,465(29.2) | 31,081(38.0) | 59,384(26.0) | 3,170(40.4) | 87,295(28.9) | 1,169(44.5) | 1,197(43.0) | 222(42.7) | 384(24.2) | 192(36.9) |
| No | 219,171(70.7) | 50,540(61.8) | 168,631(73.8) | 4,656(59.3) | 214,515(71.0) | 144(55.0) | 1,583(56.9) | 296(56.9) | 1,201(75.6) | 327(62.9) |
| Prefer not to answer | 517(0.2) | 178(0.2) | 339(0.1) | 22(0.3) | 495(0.2) | 12(0.5) | 4(0.1) | 2(0.4) | 4(0.3) | 1(0.2) |
| NA | 9(0.0) |  |  |  |  |  |  |  |  |  |
| Diabetes |  |  |  |  |  |  |  |  |  |  |
| Yes | 14,861(4.8) | 5,404(6.6) | 9,457(4.1) | 607(7.7) | 14,254(4.7) | 293(11.2) | 238(8.5) | 61(11.7) | 79(5.0) | 34(6.5) |
| No | 295,301(95.2) | 76,398(93.4) | 218,903(95.9) | 7,241(92.3) | 288,060(95.3) | 2,332(88.8) | 2,546(91.5) | 459(88.3) | 1,510(95.0) | 486(93.5) |
| Fracture History |  |  |  |  |  |  |  |  |  |  |
| Yes | 29,753(9.6) | 9,350(11.4) | 20,403(8.9) | 967(12.3) | 28,786(9.5) | 287(10.9) | 263(9.4) | 72(13.8) | 200(12.6) | 59(11.3) |
| No | 280,409(90.4) | 72,452(88.6) | 207,957(91.1) | 6,881(87.7) | 273,528(90.5) | 2,338(89.1) | 2,521(90.6) | 448(86.2) | 1,389(87.4) | 461(88.7) |
| Abbreviations: SMA, Spinal muscular atrophy and related syndromes; PD, Parkinson’s disease; AD, Alzheimer’s disease; MS, Multiple sclerosis; DANS, Disorders of autonomic nervous system; OA, Osteoarthritis; RA, Rheumatoid Arthritis; SD, standard deviation; BMI, body mass index; CHD, coronary heart disease; TDI, Townsend deprivation index. | | | | | | | | | | |

**Table S3.** Logistic regression analyses of OA/RA and incident neurodegenerative outcomes in UK Biobank

|  | Osteoarthritis (cases, n=81,802) | | | | Rheumatoid arthritis (cases, n=7,878) | | | |
| --- | --- | --- | --- | --- | --- | --- | --- | --- |
|  | Crude model (Model 1) | | Fully adjusted model (Model 2) | | Crude model (Model 1) | | Fully adjusted model (Model 2) | |
|  | OR (95%CI) | *P* | OR (95%CI) | *P* | OR (95%CI) | *P* | OR (95%CI) | *P* |
| AD | 2.17 (2.01, 2.35) | <0.001 | 1.30 (1.19, 1.40) | <0.001 | 1.66 (1.36, 2.01) | <0.001 | 1.14 (0.93, 1.38) | 0.199 |
| PD | 1.93 (1.79, 2.08) | <0.001 | 1.44 (1.33, 1.56) | <0.001 | 0.92 (0.71, 1.17) | 0.509 | 0.79 (0.61, 1.00) | 0.061 |
| DANS | 1.69 (1.41, 2.02) | <0.001 | 1.52 (1.26, 1.84) | <0.001 | 1.38 (0.82, 2.14) | 0.178 | 1.22 (0.73, 1.90) | 0.406 |
| MS | 0.95 (0.85, 1.06) | 0.359 | 1.03 (0.91, 1.12) | 0.614 | 0.19 (0.09, 0.36) | <0.001 | 0.17 (0.07, 0.33) | <0.001 |
| SMA | 1.16 (0.96, 1.40) | 0.115 | 0.96 (0.78, 1.16) | 0.647 | 1.07 (0.60, 1.74) | 0.814 | 0.98 (0.55, 1.60) | 0.931 |
| OA, Osteoarthritis; RA, Rheumatoid Arthritis; AD, Alzheimer’s disease; PD, Parkinson’s disease; DANS, Disorders of autonomic nervous system; MS, Multiple sclerosis; SMA, Spinal muscular atrophy and related syndromes; OR, odds ratio; Crude model: adjusting for Age, Sex; Fully adjusted model: adjusting for Age, Sex, TDI, Education, BMI, Smoking, Alcohol, Cancer, CHD, Diabetes and Fracture History. | | | | | | | | |

**Table S4.** Sensitivity analyses of associations between OA/RA and incident neurodegenerative outcomes.

|  | Osteoarthritis (cases, n=54,509) | | | | Rheumatoid arthritis (cases, n=3,940) | | | |
| --- | --- | --- | --- | --- | --- | --- | --- | --- |
|  | Crude model (Model 1) | | Fully adjusted model (Model 2) | | Crude model (Model 1) | | Fully adjusted model (Model 2) | |
|  | HR (95%CI) | P | HR (95%CI) | P | HR (95%CI) | P | HR (95%CI) | P |
| Sensitivity analysis: participants with recorded use of DMARDs, biologics, glucocorticoids, or NSAIDs were excluded. | | | | | | | | |
| AD | 1.21 (1.09, 1.34) | <0.001 | 1.17 (1.05, 1.29) | 0.004 | 1.76 (1.34, 2.30) | <0.001 | 1.64 (1.25, 2.15) | <0.001 |
| PD | 1.10 (0.98, 1.24) | 0.113 | 1.07 (0.95, 1.21) | 0.251 | 1.30 (0.89, 1.89) | 0.176 | 1.27 (0.87, 1.85) | 0.215 |
| DANS | 1.41 (1.05, 1.89) | 0.021 | 1.43 (1.06, 1.94) | 0.021 | 2.68 (1.32, 5.44) | 0.006 | 2.62 (1.28, 5.34) | 0.008 |
| MS | 1.07 (0.75, 1.51) | 0.720 | 1.05 (0.74, 1.47) | 0.804 | 0.38 (0.05, 2.69) | 0.331 | 0.35 (0.05, 2.52) | 0.298 |
| SMA | 1.24 (0.96, 1.60) | 0.102 | 1.26 (0.97, 1.62) | 0.085 | 2.13 (1.09, 4.13) | 0.026 | 2.14 (1.10, 4.17) | 0.026 |
| OA, Osteoarthritis; RA, Rheumatoid Arthritis; AD, Alzheimer’s disease; PD, Parkinson’s disease; DANS, Disorders of autonomic nervous system; MS, Multiple sclerosis; SMA, Spinal muscular atrophy and related syndromes; HR, hazard ratios; Crude model: adjusting for Age, Sex; Fully adjusted model: adjusting for Age, Sex, TDI, Education, BMI, Smoking, Alcohol, Cancer, CHD, Diabetes, Fracture History. | | | | | | | | |

| **Table S5.** Summary for IVs in MR analysis | | | | |
| --- | --- | --- | --- | --- |
| Causality | SNP | β | SE | P |
| RA to PD | rs10488631 | -0.2221538 | 0.16676923 | 0.18282624 |
|  | rs10818482 | 0.14829396 | 0.22703412 | 0.51364076 |
|  | rs10905284 | -0.3902439 | 0.2347561 | 0.09644509 |
|  | rs11089637 | 0.07387387 | 0.21531532 | 0.73152606 |
|  | rs11574914 | -0.2781186 | 0.21370143 | 0.19310946 |
|  | rs12460655 | 0.36134684 | 0.14645497 | 0.01361402 |
|  | rs13333054 | -0.2806851 | 0.2340628 | 0.23045524 |
|  | rs137685 | -0.0516854 | 0.2258427 | 0.81898106 |
|  | rs1516974 | -0.2427565 | 0.19342208 | 0.20945672 |
|  | rs16894540 | 0.17316342 | 0.17541229 | 0.32355466 |
|  | rs1748041 | -0.1163636 | 0.22909091 | 0.61149787 |
|  | rs17534670 | 0.09623894 | 0.23340708 | 0.68010322 |
|  | rs206769 | -0.2236155 | 0.14211076 | 0.11559628 |
|  | rs2249937 | -0.611991 | 0.33823529 | 0.07039439 |
|  | rs2261033 | -0.0732317 | 0.04448888 | 0.09974988 |
|  | rs2352593 | -0.0066934 | 0.25167336 | 0.97878217 |
|  | rs238516 | 0 | 0.25106383 | 1 |
|  | rs2476601 | -0.0729914 | 0.05350248 | 0.17248538 |
|  | rs2736340 | -0.2402659 | 0.20512821 | 0.2414797 |
|  | rs2793108 | 0.28449502 | 0.27596017 | 0.30257466 |
|  | rs2843401 | 0.0938914 | 0.20361991 | 0.64471889 |
|  | rs2930047 | 0.78571429 | 0.29649596 | 0.00804918 |
|  | rs3087243 | -0.2417219 | 0.13907285 | 0.08219403 |
|  | rs3093024 | -0.0071878 | 0.15184187 | 0.96224442 |
|  | rs3135363 | -0.0329826 | 0.04330231 | 0.44624968 |
|  | rs3184504 | 0.05269187 | 0.19129439 | 0.78297122 |
|  | rs3185010 | 0.54266212 | 0.22411832 | 0.01546427 |
|  | rs34695944 | -0.2519305 | 0.16795367 | 0.1336144 |
|  | rs3806624 | -0.5122265 | 0.23680824 | 0.03053784 |
|  | rs4239702 | -0.2799228 | 0.18339768 | 0.12693124 |
|  | rs4274624 | -0.0575365 | 0.1636953 | 0.72522441 |
|  | rs4713610 | -0.1033773 | 0.1083196 | 0.33989506 |
|  | rs478582 | -0.0807382 | 0.22145329 | 0.71542246 |
|  | rs4938573 | -0.040131 | 0.17444717 | 0.81805529 |
|  | rs548234 | -0.2831169 | 0.23376623 | 0.22585282 |
|  | rs556560 | 0.75489068 | 0.25201381 | 0.00274055 |
|  | rs6429207 | -0.2309217 | 0.2309217 | 0.31731051 |
|  | rs6679356 | 0.12111801 | 0.23498965 | 0.60626061 |
|  | rs6762816 | -0.2475138 | 0.18674033 | 0.185024 |
|  | rs678347 | -0.0706587 | 0.2994012 | 0.81343267 |
|  | rs6920220 | -0.207962 | 0.12240048 | 0.0893137 |
|  | rs706778 | 0.40553435 | 0.17938931 | 0.02378166 |
|  | rs7170151 | -0.0019569 | 0.1888454 | 0.99173191 |
|  | rs7528321 | 0.16109046 | 0.25402726 | 0.52598532 |
|  | rs7529425 | -0.6081923 | 0.23508459 | 0.00967815 |
|  | rs7553012 | -0.43769 | 0.20162107 | 0.02994219 |
|  | rs7579944 | -0.5153129 | 0.25432756 | 0.04274655 |
|  | rs8026898 | -0.0123636 | 0.13963636 | 0.92944617 |
|  | rs9653442 | -0.3047035 | 0.17177914 | 0.07609466 |
|  | rs9909593 | 0.21006289 | 0.21132075 | 0.32019968 |
|  | rs9979383 | 0.26231527 | 0.28448276 | 0.35648793 |
|  | rs998731 | -0.1413748 | 0.29831388 | 0.63556192 |
| MS to RA | rs10063294 | -0.077740714 | 0.127212077 | 0.541126024 |
|  | rs1014486 | 0.098020556 | 0.121811953 | 0.421000035 |
|  | rs10209615 | -0.141442716 | 0.11411079 | 0.215152632 |
|  | rs1026916 | 0.233700724 | 0.102581506 | 0.022714933 |
|  | rs1077667 | -0.207425162 | 0.109309768 | 0.057750065 |
|  | rs11164820 | 0.247742903 | 0.101317826 | 0.014476964 |
|  | rs11666263 | 0.31509234 | 0.14004104 | 0.024448945 |
|  | rs1177287 | 0.399387926 | 0.137620418 | 0.003706727 |
|  | rs12048904 | 0.028413665 | 0.121231638 | 0.814693865 |
|  | rs12148050 | 0.204496917 | 0.141032357 | 0.147058519 |
|  | rs12187530 | 0.130787473 | 0.093637724 | 0.162492033 |
|  | rs12365699 | 0.831976849 | 0.127996438 | 8.032E-11 |
|  | rs12466022 | -0.004959579 | 0.117376713 | 0.966296597 |
|  | rs1250550 | 0.242368032 | 0.122496478 | 0.047864279 |
|  | rs1292061 | -0.002914942 | 0.133115684 | 0.982529472 |
|  | rs12928822 | 0.313386629 | 0.127788723 | 0.014191433 |
|  | rs1321859 | 0.355993956 | 0.128862395 | 0.005734476 |
|  | rs13327021 | 0.498324449 | 0.117201743 | 2.1201E-05 |
|  | rs140522 | -0.067814388 | 0.132012008 | 0.60746271 |
|  | rs17040773 | -0.089456367 | 0.085965387 | 0.298056989 |
|  | rs17066096 | 0.005166357 | 0.111445694 | 0.963025218 |
|  | rs17124032 | 0.279516427 | 0.109777079 | 0.010889701 |
|  | rs1738074 | 0.568016953 | 0.114306817 | 6.7219E-07 |
|  | rs1828668 | -0.315705115 | 0.135591299 | 0.019893091 |
|  | rs1860545 | -0.082379391 | 0.120136612 | 0.492893296 |
|  | rs2104286 | 0.320117835 | 0.071682828 | 7.97875E-06 |
|  | rs2248461 | -0.035138984 | 0.121137024 | 0.771757839 |
|  | rs2255214 | 0.262994151 | 0.105363587 | 0.01255803 |
|  | rs2517830 | -0.054440707 | 0.026259635 | 0.038156393 |
|  | rs2546890 | 0.192335639 | 0.108562783 | 0.076452086 |
|  | rs2857700 | -0.304995673 | 0.027110726 | 2.31592E-29 |
|  | rs28743267 | -0.367652465 | 0.033170422 | 1.50447E-28 |
|  | rs354033 | -0.191743009 | 0.136165325 | 0.159082731 |
|  | rs4074156 | -0.011322539 | 0.139200629 | 0.935171792 |
|  | rs41479249 | -0.517038778 | 0.129638755 | 6.65473E-05 |
|  | rs4410871 | 0.461639705 | 0.139474124 | 0.000933429 |
|  | rs4648356 | 0.575086806 | 0.099012587 | 6.31408E-09 |
|  | rs4739134 | 0.366356574 | 0.138103002 | 0.007983241 |
|  | rs4794001 | -0.21910852 | 0.119428249 | 0.066558207 |
|  | rs4902647 | -0.453937031 | 0.122213816 | 0.000203778 |
|  | rs5755694 | 0.269095609 | 0.127466341 | 0.034762763 |
|  | rs6065926 | -0.793080745 | 0.112228407 | 1.587E-12 |
|  | rs650258 | -0.15549061 | 0.10990174 | 0.157122799 |
|  | rs6677309 | 0.003757068 | 0.092987433 | 0.967771009 |
|  | rs669607 | 0.323733139 | 0.094057601 | 0.000577728 |
|  | rs6944568 | 0.084024199 | 0.162861719 | 0.605907794 |
|  | rs701006 | 0.613890255 | 0.114171292 | 7.57695E-08 |
|  | rs7162687 | 0.51649888 | 0.168488955 | 0.002173237 |
|  | rs7200786 | 0.084670932 | 0.078623009 | 0.281514633 |
|  | rs7238078 | 0.163169733 | 0.137357572 | 0.234865141 |
|  | rs7554511 | -0.482634926 | 0.1149519 | 2.68592E-05 |
|  | rs7595037 | -0.292817307 | 0.122328283 | 0.016679349 |
|  | rs7750586 | 0.208199481 | 0.111419254 | 0.061676656 |
|  | rs7855251 | -0.173464476 | 0.135320455 | 0.199885027 |
|  | rs7923866 | 0.091895417 | 0.128455959 | 0.474371416 |
|  | rs8108669 | -0.020952534 | 0.12974454 | 0.871706949 |
|  | rs874628 | 0.346157282 | 0.107197094 | 0.001241515 |
|  | rs9268541 | 1.810365201 | 0.108086692 | 5.73966E-63 |
|  | rs9591325 | 0.297599427 | 0.132789618 | 0.025017348 |

**Table S6.** MR-PRESSO abnormal SNP report

| Exposure | Outcome | Global Test RSSobs | Global Test P value | Distortion Test  Outliers Indices | Distortion Test Distortion Coefficient beta. exposure | Distortion Test P value |
| --- | --- | --- | --- | --- | --- | --- |
| The forward MR analyses | | | | | | |
| OA | AD | 51.75867171 | 0.012 | rs2929305 | -53.06911 | 0.714 |
|  | PD | 23.57278604 | 0.643 | - | - | - |
|  | MS | 104.6536 | <0.001 | rs12693984、rs1800562、rs2695478 | 37.18125 | 0.732 |
| RA | AD | 93.4487416 | 0.005 | rs9909593 | 55.03039 | 0.869 |
|  | PD | 91.81976228 | 0.003 | rs556560 | 7.05102 | 0.789 |
|  | MS | 1333.647 | <0.001 | rs13333054、rs17534670、rs206769、rs2249937、rs2261033、rs2352593、rs238516、rs2843401、rs3135363、rs3184504、rs34695944、rs3806624、rs4239702、rs4713610、rs4938573、rs6920220、rs706778、rs7528321、rs9909593 | 319.7247 | 0.008 |
| The reverse MR analyses | | | | | | |
| AD | OA | 61.12695611 | 0.018 | No significant outliers | NA | NA |
|  | RA | 3528.251 | <0.001 | rs11495981、rs1693551、rs3997872、rs7254892 | -533.0886 | <0.001 |
| PD | OA | 29.2882 | 29.2882 | - | - | - |
|  | RA | 3575.516 | <0.001 | rs17761207、rs2157990、rs2230288、rs356221、rs3997872、rs6430538、rs6599389、rs9917256 | -443.2628 | <0.001 |
| MS | OA | 87.344787 | 0.006 | rs7299943 | -121.6943044 | 0.166 |
|  | RA | 1040.419924 | <0.001 | rs12365699、rs13327021、rs1738074、rs2104286、rs2857700、rs28743267、rs41479249、rs4648356、rs6065926、rs669607、rs701006、rs7162687、rs7554511、rs874628、  rs9268541 | -220.495 | 0.008 |

**Table S7.** Summary for RA TWAS results in tibial nerve

| Ensembl ID | Gene | Chromosome | Position | HSQ | BEST GWAS ID | BEST GWAS Z | EQTL ID | EQTL R2 | EQTL Z | EQTL GWAS Z | NSNP | NWGT | MODEL | MODELCV R2 | MODELCV PV | TWAS Z | TWAS P | Tissue |
| --- | --- | --- | --- | --- | --- | --- | --- | --- | --- | --- | --- | --- | --- | --- | --- | --- | --- | --- |
| ENSG00000064012.21 | CASP8 | 2 | 201233442-201233443 | 0.146 | rs16837131 | 5.54 | rs16837131 | 0.106 | -6.96 | 5.5381 | 350 | 1 | top1 | 0.11 | 1.60E-12 | -5.538095 | 3.06E-08 | Nerve tibial |
| ENSG00000081026.18 | MAGI3 | 1 | 113390748-113390749 | 0.235 | rs6679677 | 27.65 | rs4839315 | 0.0821 | -7.14 | -0.91411 | 367 | 34 | enet | 0.18 | 2.70E-20 | -8.29 | 1.13E-16 | Nerve tibial |
| ENSG00000101017.13 | CD40 | 20 | 46118271-46118272 | 0.1907 | rs4239702 | -7.19 | rs1883832 | 0.144758 | -8.03 | -7.00676 | 501 | 1 | top1 | 0.14 | 9.40E-17 | 7.00676 | 2.44E-12 | Nerve tibial |
| ENSG00000105397.13 | TYK2 | 19 | 10380675-10380676 | 0.1498 | rs12460655 | -7 | rs280519 | 0.070664 | -5.9 | 4.92424 | 377 | 1 | top1 | 0.071 | 9.60E-09 | -4.92424 | 8.47E-07 | Nerve tibial |
| ENSG00000108306.11 | FBXL20 | 17 | 39402522-39402523 | 0.1034 | rs9909593 | 6.21 | rs12453682 | 0.039922 | -5.68 | -4.20979 | 330 | 6 | lasso | 0.064 | 4.70E-08 | 4.96895 | 6.73E-07 | Nerve tibial |
| ENSG00000117122.13 | MFAP2 | 1 | 16980834-16980835 | 0.2094 | rs1748041 | 6.07 | rs761422 | 0.124 | 8.49 | 4.29677 | 344 | 15 | enet | 0.17 | 4.20E-19 | 4.62 | 3.79E-06 | Nerve tibial |
| ENSG00000125637.15 | PSD4 | 2 | 113157324-113157325 | 0.4609 | rs17669228 | 4.34 | rs12711765 | 0.163 | 9.26 | -3.58599 | 451 | 31 | enet | 0.3 | 2.90E-36 | -5.52521 | 3.29E-08 | Nerve tibial |
| ENSG00000128604.19 | IRF5 | 7 | 128937611-128937612 | 0.2152 | rs10488631 | 8.33 | rs3807306 | 0.157 | -9.23 | -8.23077 | 332 | 15 | enet | 0.2 | 9.10E-23 | 7.88798 | 3.07E-15 | Nerve tibial |
| ENSG00000133103.16 | COG6 | 13 | 39655626-39655627 | 0.3846 | rs12875311 | -7.89 | rs1028671 | 0.360681 | -12.64 | -5.1446 | 525 | 1 | top1 | 0.36 | 1.70E-44 | 5.1446 | 2.68E-07 | Nerve tibial |
| ENSG00000134824.13 | FADS2 | 11 | 61816255-61816256 | 0.4246 | rs968567 | -5.3 | rs968567 | 0.293 | 11.35 | -5.30114 | 414 | 4 | lasso | 0.29 | 4.80E-35 | -5.07274 | 3.92E-07 | Nerve tibial |
| ENSG00000136573.12 | BLK | 8 | 11494000-11494001 | 0.0739 | rs2736340 | 6.62 | rs12680762 | 0.0265 | -4.33 | 5.05988 | 630 | 1 | top1 | 0.026 | 0.00037 | -5.05988 | 4.20E-07 | Nerve tibial |
| ENSG00000138386.16 | NAB1 | 2 | 190646745-190646746 | 0.0927 | rs4274624 | 8.28 | rs3771317 | 0.0406 | 5.07 | 4.55191 | 371 | 5 | lasso | 0.064 | 4.50E-08 | 5.10144 | 3.37E-07 | Nerve tibial |
| ENSG00000139531.12 | SUOX | 12 | 55997179-55997180 | 0.4939 | rs11171710 | 5.86 | rs705700 | 0.437 | -13.98 | -5.82171 | 292 | 18 | enet | 0.47 | 9.20E-63 | 6.09238 | 1.11E-09 | Nerve tibial |
| ENSG00000141736.13 | ERBB2 | 17 | 39688107-39688108 | 0.0868 | rs9909593 | 6.21 | rs2941504 | 0.101019 | 7.08 | -4.90647 | 321 | 321 | susie | 0.11 | 1.10E-12 | -4.97349 | 6.58E-07 | Nerve tibial |
| ENSG00000141744.3 | PNMT | 17 | 39667980-39667981 | 0.0896 | rs9909593 | 6.21 | rs1877031 | 0.059401 | 5.65 | -4.47794 | 323 | 323 | susie | 0.066 | 2.80E-08 | -4.64699 | 3.37E-06 | Nerve tibial |
| ENSG00000145725.19 | PPIP5K2 | 5 | 103120151-103120152 | 0.2076 | rs556560 | -6.534 | rs34823 | 0.163188 | -9 | -4.2 | 318 | 11 | lasso | 0.19 | 5.10E-22 | 4.99573 | 5.86E-07 | Nerve tibial |
| ENSG00000149485.18 | FADS1 | 11 | 61829317-61829318 | 0.2084 | rs968567 | -5.3 | rs174555 | 0.172 | -8.84 | -4.74101 | 421 | 421 | susie | 0.17 | 6.90E-20 | 4.74539 | 2.08E-06 | Nerve tibial |
| ENSG00000156860.15 | FBRS | 16 | 30658430-30658431 | 0.0617 | rs8058578 | 5.17 | rs13338946 | 0.041018 | -4.42 | 5.05921 | 194 | 1 | top1 | 0.041 | 1.20E-05 | -5.05921 | 4.21E-07 | Nerve tibial |
| ENSG00000161180.10 | CCDC116 | 22 | 21632715-21632716 | 0.1776 | rs11089637 | 6.34 | rs2298429 | 0.202764 | -9.47 | 5.68902 | 298 | 21 | enet | 0.21 | 5.70E-24 | -4.99326 | 5.94E-07 | Nerve tibial |
| ENSG00000161395.13 | PGAP3 | 17 | 39696796-39696797 | 0.1196 | rs9909593 | 6.21 | rs903502 | 0.113221 | 8.18 | -5.15328 | 319 | 319 | susie | 0.13 | 1.70E-15 | -5.1682 | 2.36E-07 | Nerve tibial |
| ENSG00000166908.17 | PIP4K2C | 12 | 57591173-57591174 | 0.0308 | rs238516 | -5.47 | rs1021469 | 0.0123 | -3.92 | -4.23077 | 339 | 339 | susie | 0.02 | 0.0018 | 4.94685 | 7.54E-07 | Nerve tibial |
| ENSG00000173517.10 | PEAK1 | 15 | 77420143-77420144 | 0.1166 | rs17384809 | -4.84 | rs11856084 | 0.128 | -7.72 | -4.8314 | 326 | 326 | susie | 0.13 | 3.40E-15 | 4.69542 | 2.66E-06 | Nerve tibial |
| ENSG00000178093.13 | TSSK6 | 19 | 19515684-19515685 | 0.1224 | rs11555053 | 5.15 | rs1036215 | 0.13143 | 7.9 | 4.89904 | 355 | 1 | top1 | 0.13 | 2.90E-15 | 4.89904 | 9.63E-07 | Nerve tibial |
| ENSG00000184602.5 | SNN | 16 | 11668413-11668414 | 0.1188 | rs4439779 | -6.18 | rs933573 | 0.047796 | -5.42 | -5.46212 | 477 | 477 | susie | 0.063 | 5.90E-08 | 5.55238 | 2.82E-08 | Nerve tibial |
| ENSG00000185651.14 | UBE2L3 | 22 | 21567546-21567547 | 0.0404 | rs11089637 | 6.34 | rs4820091 | 0.026073 | 4.69 | 5.56098 | 286 | 286 | susie | 0.029 | 0.00019 | 5.78567 | 7.22E-09 | Nerve tibial |
| ENSG00000187664.8 | HAPLN4 | 19 | 19262795-19262796 | 0.1611 | rs11555053 | 5.15 | rs2074296 | 0.149994 | 8.31 | 4.59615 | 352 | 1 | top1 | 0.15 | 2.40E-17 | 4.59615 | 4.30E-06 | Nerve tibial |
| ENSG00000196118.11 | CFAP119 | 16 | 30762709-30762710 | 0.1242 | rs8058578 | 5.17 | rs8058578 | 0.054359 | -6.29 | 5.1745 | 208 | 208 | susie | 0.076 | 2.60E-09 | -5.06126 | 4.16E-07 | Nerve tibial |
| ENSG00000197728.9 | RPS26 | 12 | 56041852-56041853 | 0.8589 | rs11171710 | 5.86 | rs10876864 | 0.774 | 18.4 | -5.125 | 289 | 19 | enet | 0.79 | 4.40E-148 | -5.12823 | 2.92E-07 | Nerve tibial |
| ENSG00000204084.12 | INPP5B | 1 | 37947056-37947057 | 0.4965 | rs7553012 | -6.33 | rs4653329 | 0.308 | 11.7 | 4.87742 | 454 | 10 | lasso | 0.39 | 9.40E-49 | 5.34 | 9.40E-08 | Nerve tibial |
| ENSG00000215912.12 | TTC34 | 1 | 2801716-2801717 | 0.1321 | rs2843401 | -6.5 | rs4648356 | 0.0626 | 5.71 | -5.80822 | 367 | 1 | top1 | 0.063 | 6.60E-08 | -5.81 | 6.31E-09 | Nerve tibial |
| ENSG00000220201.7 | ZGLP1 | 19 | 10309879-10309880 | 0.1442 | rs12460655 | -7 | rs12720356 | 0.062689 | -5.46 | -5.74603 | 387 | 33 | enet | 0.088 | 1.30E-10 | 4.70311 | 2.56E-06 | Nerve tibial |
| ENSG00000228549.3 | LINC03126 | 1 | 16870944-16870945 | 0.0525 | rs1748041 | 6.07 | rs2235925 | 0.0275 | 4.04 | -4.82317 | 328 | 1 | top1 | 0.028 | 0.00029 | -4.82 | 1.41E-06 | Nerve tibial |
| ENSG00000234255.8 | - | 2 | 65456570-65456571 | 0.1614 | rs7584295 | -7.17 | rs6755128 | 0.103 | 7.26 | -5.57664 | 526 | 526 | susie | 0.11 | 2.80E-13 | -5.71385 | 1.10E-08 | Nerve tibial |
| ENSG00000246575.2 | - | 2 | 85315040-85315041 | 0.1867 | rs10460587 | 4.65 | rs1053560 | 0.116 | -7.23 | 4.37302 | 446 | 446 | susie | 0.12 | 3.20E-14 | -4.696175 | 2.65E-06 | Nerve tibial |
| ENSG00000267100.1 | ILF3-DT | 19 | 10653843-10653844 | 0.1692 | rs12460655 | -7 | rs8099926 | 0.033491 | -5.89 | 4.99301 | 370 | 14 | enet | 0.12 | 1.80E-13 | -5.45985 | 4.77E-08 | Nerve tibial |
| ENSG00000267673.6 | FDX2 | 19 | 10316014-10316015 | 0.1072 | rs12460655 | -7 | rs12720356 | 0.048387 | -4.95 | -5.74603 | 386 | 4 | lasso | 0.07 | 1.10E-08 | 7.2707 | 3.58E-13 | Nerve tibial |
| ENSG00000270424.1 | - | 16 | 28750594-28750595 | 0.0994 | rs7500321 | 4.75 | rs28698667 | 0.059523 | 6.73 | 3.68217 | 197 | 15 | enet | 0.078 | 1.70E-09 | 4.80024 | 1.58E-06 | Nerve tibial |
| ENSG00000271913.5 | TAGAP-AS1 | 6 | 158988177-158988178 | 0.1896 | rs654690 | -6.75 | rs9295089 | 0.062 | -5.42 | -4.6729 | 544 | 1 | top1 | 0.062 | 7.70E-08 | 4.6729 | 2.97E-06 | Nerve tibial |
| ENSG00000273342.1 | - | 22 | 21640843-21640844 | 0.0428 | rs11089637 | 6.34 | rs2298429 | 0.029068 | -4.15 | 5.68902 | 300 | 300 | susie | 0.033 | 8.40E-05 | -5.78908 | 7.08E-09 | Nerve tibial |
| ENSG00000273890.1 | - | 12 | 56010573-56010574 | 0.066 | rs11171710 | 5.86 | rs705700 | 0.00211 | 4.16 | -5.82171 | 284 | 3 | lasso | 0.004 | 0.097 | -5.7137 | 1.11E-08 | Nerve tibial |
| ENSG00000275106.1 | - | 7 | 128953315-128953316 | 0.0766 | rs10488631 | 8.33 | rs6969930 | 0.0666 | 5.71 | -5.77863 | 330 | 1 | top1 | 0.067 | 2.50E-08 | -5.77863 | 7.53E-09 | Nerve tibial |

**Table S8.** Summary for PD TWAS results in tibial nerve

| Esembl ID | Gene | Chromosome | Position | HSQ | BEST GWAS ID | BEST GWAS Z | EQTL ID | EQTL R2 | EQTL Z | EQTL GWAS Z | NSNP | NWGT | MODEL | MODELCV R2 | MODELCV PV | TWAS Z | TWAS P | Tissue |
| --- | --- | --- | --- | --- | --- | --- | --- | --- | --- | --- | --- | --- | --- | --- | --- | --- | --- | --- |
| ENSG00000011295.15 | TTC19 | 17 | 15999379-15999380 | 0.1351 | rs4566208 | 5.43 | rs2779211 | 0.163197 | -8.98 | 4.97076 | 275 | 275 | susie | 0.17 | 1.60E-19 | -4.65179 | 3.29E-06 | Nerve tibial |
| ENSG00000035499.12 | DEPDC1B | 5 | 60700189-60700190 | 0.0589 | rs16878547 | 7.02 | rs16878547 | 0.050581 | -4.83 | 7.01901 | 319 | 1 | top1 | 0.051 | 1.20E-06 | -7.01901 | 2.23E-12 | Nerve tibial |
| ENSG00000073969.18 | NSF | 17 | 46590668-46590669 | 0.0791 | rs199452 | -9.43 | rs17692129 | 0.016566 | 3.82 | 3.27586 | 146 | 4 | lasso | 0.019 | 0.0021 | 6.67486 | 2.47E-11 | Nerve tibial |
| ENSG00000099365.10 | STX1B | 16 | 31010660-31010661 | 0.2046 | rs8050588 | -6.4 | rs12445568 | 0.235144 | 10.55 | -5.48594 | 231 | 231 | susie | 0.24 | 7.50E-28 | -5.38607 | 7.20E-08 | Nerve tibial |
| ENSG00000103496.14 | STX4 | 16 | 31032888-31032889 | 0.1198 | rs8050588 | -6.4 | rs729482 | 0.104395 | 7.31 | -5.26087 | 240 | 240 | susie | 0.11 | 9.70E-13 | -5.96075 | 2.51E-09 | Nerve tibial |
| ENSG00000103510.19 | KAT8 | 16 | 31115753-31115754 | 0.2236 | rs8050588 | -6.4 | rs9936329 | 0.20799 | -9.65 | 5.29775 | 243 | 243 | susie | 0.25 | 3.40E-29 | -5.69904 | 1.20E-08 | Nerve tibial |
| ENSG00000108379.9 | WNT3 | 17 | 46833057-46833058 | 0.1985 | rs199452 | -9.43 | rs199520 | 0.154851 | 8.47 | -8.04846 | 256 | 256 | susie | 0.16 | 3.20E-18 | -8.21363 | 2.15E-16 | Nerve tibial |
| ENSG00000117280.12 | RAB29 | 1 | 205775459-205775460 | 0.3017 | rs2153904 | 6.235 | rs708723 | 0.176 | -9.61 | -5.72515 | 428 | 43 | enet | 0.24 | 2.30E-28 | 5.06288 | 4.13E-07 | Nerve tibial |
| ENSG00000120088.14 | CRHR1 | 17 | 45784279-45784280 | 0.1089 | rs17689918 | -9.32 | rs17689882 | 0.080441 | 6.46 | -9.2963 | 33 | 17 | enet | 0.081 | 7.30E-10 | -8.64378 | 5.44E-18 | Nerve tibial |
| ENSG00000127415.12 | IDUA | 4 | 986996-986997 | 0.3453 | rs4690326 | 6.7 | rs4690220 | 0.167967 | 8.98 | -5.33708 | 400 | 7 | lasso | 0.19 | 5.40E-22 | -5.58707 | 2.31E-08 | Nerve tibial |
| ENSG00000127419.16 | TMEM175 | 4 | 932386-932387 | 0.1169 | rs4690326 | 6.7 | rs4690336 | 0.046428 | -5.39 | 5.66071 | 417 | 1 | top1 | 0.046 | 3.20E-06 | -5.66071 | 1.51E-08 | Nerve tibial |
| ENSG00000136235.15 | GPNMB | 7 | 23235966-23235967 | 0.0931 | rs466225 | -5.86 | rs199355 | 0.0627 | -5.61 | -5.7124 | 413 | 1 | top1 | 0.063 | 6.60E-08 | 5.7124 | 1.11E-08 | Nerve tibial |
| ENSG00000136243.16 | NUP42 | 7 | 23181826-23181827 | 0.1638 | rs466225 | -5.86 | rs6955115 | 0.289 | -11.43 | -4.92818 | 426 | 1 | top1 | 0.29 | 1.90E-34 | 4.92818 | 8.30E-07 | Nerve tibial |
| ENSG00000138722.9 | MMRN1 | 4 | 89879531-89879532 | 0.2941 | rs2736990 | 11.83 | rs1866995 | 0.198744 | 9.53 | 7.47344 | 388 | 388 | susie | 0.23 | 3.50E-26 | 6.30102 | 2.96E-10 | Nerve tibial |
| ENSG00000145214.13 | DGKQ | 4 | 986894-986895 | 0.5045 | rs4690326 | 6.7 | rs4690220 | 0.379014 | 12.91 | -5.33708 | 400 | 400 | susie | 0.43 | 4.90E-56 | -6.73893 | 1.60E-11 | Nerve tibial |
| ENSG00000152128.13 | TMEM163 | 2 | 134718999-134719000 | 0.0782 | rs6430538 | -7 | rs6430538 | 0.0727 | -5.8 | -7 | 433 | 1 | top1 | 0.073 | 5.90E-09 | 7 | 2.56E-12 | Nerve tibial |
| ENSG00000156860.15 | FBRS | 16 | 30658430-30658431 | 0.0617 | rs8050588 | -6.4 | rs13338946 | 0.041018 | -4.42 | -4.87374 | 194 | 1 | top1 | 0.041 | 1.20E-05 | 4.87374 | 1.10E-06 | Nerve tibial |
| ENSG00000167395.10 | ZNF646 | 16 | 31074421-31074422 | 0.076 | rs8050588 | -6.4 | rs7197717 | 0.052182 | 5.38 | -4.92818 | 242 | 1 | top1 | 0.052 | 8.10E-07 | -4.92818 | 8.30E-07 | Nerve tibial |
| ENSG00000170425.3 | ADORA2B | 17 | 15944916-15944917 | 0.315 | rs2078050 | 5.42 | rs1045599 | 0.406679 | 13.42 | 5.11561 | 292 | 292 | susie | 0.41 | 1.00E-51 | 5.11074 | 3.21E-07 | Nerve tibial |
| ENSG00000176681.14 | LRRC37A | 17 | 46292732-46292733 | 0.6524 | rs199452 | -9.43 | rs8072451 | 0.329008 | 12.1 | -9.27778 | 78 | 28 | enet | 0.46 | 5.60E-61 | -7.38451 | 1.53E-13 | Nerve tibial |
| ENSG00000178950.16 | GAK | 4 | 932372-932373 | 0.2135 | rs4690326 | 6.7 | rs2061846 | 0.090244 | 6.57 | 5.56054 | 417 | 1 | top1 | 0.09 | 8.30E-11 | 5.56054 | 2.69E-08 | Nerve tibial |
| ENSG00000184445.11 | KNTC1 | 12 | 122527245-122527246 | 0.1674 | rs11060180 | -4.98 | rs1499681 | 0.0205 | 5.7 | -3.01056 | 311 | 10 | enet | 0.076 | 2.40E-09 | -4.887995 | 1.02E-06 | Nerve tibial |
| ENSG00000185829.17 | ARL17A | 17 | 46579681-46579682 | 0.4714 | rs199452 | -9.43 | rs17692129 | 0.129952 | -7.63 | 3.27586 | 142 | 142 | susie | 0.24 | 1.70E-27 | -4.97512 | 6.52E-07 | Nerve tibial |
| ENSG00000188906.14 | LRRK2 | 12 | 40196743-40196744 | 0.2855 | rs11174631 | 4.91 | rs11176052 | 0.124 | 8.26 | 2.10417 | 540 | 47 | enet | 0.24 | 2.90E-28 | 5.301321 | 1.15E-07 | Nerve tibial |
| ENSG00000196118.11 | CFAP119 | 16 | 30762709-30762710 | 0.1242 | rs8050588 | -6.4 | rs8058578 | 0.054359 | -6.29 | -4.73367 | 208 | 208 | susie | 0.076 | 2.60E-09 | 4.77965 | 1.76E-06 | Nerve tibial |
| ENSG00000198648.10 | STK39 | 2 | 168248140-168248141 | 0.1265 | rs9917256 | 6.07 | rs7569435 | 0.0279 | -4.8 | 4.4734 | 496 | 496 | susie | 0.035 | 4.70E-05 | -4.88603 | 1.03E-06 | Nerve tibial |
| ENSG00000204650.14 | LINC02210 | 17 | 45620327-45620328 | 0.6204 | rs17689918 | -9.32 | rs17689471 | 0.716122 | 17.73 | -9.31227 | 89 | 89 | susie | 0.72 | 2.80E-122 | -9.28031 | 1.69E-20 | Nerve tibial |
| ENSG00000204652.6 | RPS26P8 | 17 | 45608570-45608571 | 0.2073 | rs17689918 | -9.32 | rs8072451 | 0.296118 | 11.56 | -9.27778 | 102 | 102 | susie | 0.3 | 1.10E-35 | -9.28072 | 1.68E-20 | Nerve tibial |
| ENSG00000206053.12 | JPT2 | 16 | 1678255-1678256 | 0.3313 | rs8055439 | -4.29 | rs8058190 | 0.118233 | 7.49 | 3.24215 | 476 | 64 | enet | 0.13 | 8.80E-15 | 4.71101 | 2.46E-06 | Nerve tibial |
| ENSG00000214401.4 | KANSL1-AS1 | 17 | 46193575-46193576 | 0.4008 | rs17689918 | -9.32 | rs17689882 | 0.580074 | 15.95 | -9.2963 | 28 | 16 | enet | 0.59 | 7.50E-87 | -9.37709 | 6.78E-21 | Nerve tibial |
| ENSG00000214425.7 | LRRC37A4P | 17 | 45550334-45550335 | 0.6085 | rs17689918 | -9.32 | rs1876829 | 0.640553 | -16.78 | -9.26667 | 134 | 14 | lasso | 0.64 | 1.20E-99 | 9.28854 | 1.56E-20 | Nerve tibial |
| ENSG00000214846.4 | - | 4 | 15731626-15731627 | 0.1839 | rs4698412 | -7.49 | rs4698412 | 0.062637 | 6.49 | -7.4881 | 549 | 549 | susie | 0.15 | 2.70E-17 | -8.03869 | 9.08E-16 | Nerve tibial |
| ENSG00000226816.2 | - | 7 | 23206012-23206013 | 0.1211 | rs466225 | -5.86 | rs1990365 | 0.0915 | 7.24 | -5.57459 | 430 | 430 | susie | 0.11 | 8.50E-13 | -5.59845 | 2.16E-08 | Nerve tibial |
| ENSG00000230042.1 | AK3P3 | 7 | 23129177-23129178 | 0.0758 | rs466225 | -5.86 | rs10254544 | 0.061 | -6.04 | -4.87293 | 429 | 429 | susie | 0.07 | 1.20E-08 | 5.16181 | 2.45E-07 | Nerve tibial |
| ENSG00000230658.1 | KLHL7-DT | 7 | 23105702-23105703 | 0.5426 | rs466225 | -5.86 | rs7787110 | 0.677 | 17.24 | -5 | 428 | 8 | lasso | 0.68 | 1.80E-109 | -5.03489 | 4.78E-07 | Nerve tibial |
| ENSG00000232300.1 | FAM215B | 17 | 46562794-46562795 | 0.2151 | rs199452 | -9.43 | rs17692129 | 0.09451 | -7.1 | 3.27586 | 135 | 20 | enet | 0.15 | 2.00E-17 | -6.48744 | 8.73E-11 | Nerve tibial |
| ENSG00000232748.3 | - | 16 | 31056459-31056460 | 0.0324 | rs8050588 | -6.4 | rs9936329 | 0.026685 | 3.93 | 5.29775 | 241 | 1 | top1 | 0.027 | 0.00035 | 5.29775 | 1.17E-07 | Nerve tibial |
| ENSG00000238083.7 | LRRC37A2 | 17 | 46511510-46511511 | 0.5861 | rs199452 | -9.43 | rs169201 | 0.502118 | 14.94 | -8.9249 | 135 | 135 | susie | 0.56 | 1.60E-80 | -8.45862 | 2.71E-17 | Nerve tibial |
| ENSG00000260911.2 | - | 16 | 31043149-31043150 | 0.2606 | rs8050588 | -6.4 | rs7197717 | 0.358125 | 12.77 | -4.92818 | 241 | 241 | susie | 0.37 | 8.00E-46 | -5.02627 | 5.00E-07 | Nerve tibial |
| ENSG00000261575.2 | - | 17 | 46267036-46267037 | 0.0904 | rs199452 | -9.43 | rs17689882 | 0.169718 | 8.71 | -9.2963 | 65 | 1 | top1 | 0.17 | 1.40E-19 | -9.2963 | 1.45E-20 | Nerve tibial |
| ENSG00000262500.1 | MAPK8IP1P1 | 17 | 46243605-46243606 | 0.1604 | rs199452 | -9.43 | rs199439 | 0.340353 | 12.54 | -8.94862 | 58 | 11 | lasso | 0.36 | 2.60E-44 | -9.28958 | 1.55E-20 | Nerve tibial |
| ENSG00000262539.1 | - | 17 | 46260605-46260606 | 0.4033 | rs199452 | -9.43 | rs169201 | 0.580748 | 15.96 | -8.9249 | 65 | 1 | top1 | 0.58 | 1.50E-84 | -8.9249 | 4.46E-19 | Nerve tibial |
| ENSG00000262766.1 | - | 16 | 31118077-31118078 | 0.0563 | rs8050588 | -6.4 | rs4889606 | 0.038316 | -4.4 | 4.82584 | 244 | 1 | top1 | 0.038 | 2.20E-05 | -4.82584 | 1.39E-06 | Nerve tibial |
| ENSG00000263503.1 | MAPK8IP1P2 | 17 | 45602339-45602340 | 0.3647 | rs17689918 | -9.32 | rs8072451 | 0.518694 | 15.12 | -9.27778 | 107 | 6 | lasso | 0.52 | 1.40E-71 | -9.26574 | 1.94E-20 | Nerve tibial |
| ENSG00000264070.1 | DND1P1 | 17 | 45585870-45585871 | 0.3886 | rs17689918 | -9.32 | rs17689471 | 0.503675 | 14.94 | -9.31227 | 116 | 116 | susie | 0.51 | 2.80E-69 | -9.2802 | 1.69E-20 | Nerve tibial |
| ENSG00000266918.1 | - | 17 | 45531576-45531577 | 0.1123 | rs17689918 | -9.32 | rs17689882 | 0.079097 | 6.05 | -9.2963 | 141 | 12 | lasso | 0.083 | 4.50E-10 | -9.11515 | 7.86E-20 | Nerve tibial |
| ENSG00000278133.1 | - | 16 | 31122234-31122235 | 0.1659 | rs8050588 | -6.4 | rs1060506 | 0.081213 | -6.26 | -4.61521 | 246 | 1 | top1 | 0.081 | 7.50E-10 | 4.61521 | 3.93E-06 | Nerve tibial |
| ENSG00000280022.1 | - | 17 | 45592620-45592621 | 0.297 | rs17689918 | -9.32 | rs17689471 | 0.393472 | 13.22 | -9.31227 | 111 | 111 | susie | 0.4 | 3.10E-50 | -9.2807 | 1.68E-20 | Nerve tibial |

**Table S9.** Summary for pleiotropic genes of Parkinson's disease in 21 tissues

| Ensembl ID | Chromosome | No. SNPs | Lead SNP | PP H4 of Genes | Tissue | Disease |
| --- | --- | --- | --- | --- | --- | --- |
| ENSG00000099377 | 16 | 6259 | rs12934900 | 0.959 | Adipose_Subcutaneous | PD |
| ENSG00000136243 | 7 | 6267 | rs858295 | 0.959 | Adipose_Subcutaneous | PD |
| ENSG00000188906 | 12 | 6256 | rs75505347 | 0.997 | Adipose_Subcutaneous | PD |
| ENSG00000226816 | 7 | 6253 | rs858295 | 0.979 | Adipose_Subcutaneous | PD |
| ENSG00000226816 | 7 | 6256 | rs858295 | 0.967 | Adipose_Visceral_Omentum | PD |
| ENSG00000099377 | 16 | 5833 | rs12934900 | 0.952 | Adrenal_Gland | PD |
| ENSG00000226816 | 7 | 6616 | rs858295 | 0.978 | Adrenal_Gland | PD |
| ENSG00000004468 | 4 | 6616 | rs4698412 | 0.996 | Brain_Caudate_basal_ganglia | PD |
| ENSG00000152128 | 2 | 6616 | rs6741007 | 0.973 | Brain_Caudate_basal_ganglia | PD |
| ENSG00000099365 | 16 | 2899 | rs12934900 | 0.957 | Brain_Cerebellar_Hemisphere | PD |
| ENSG00000136243 | 7 | 2924 | rs858295 | 0.979 | Brain_Cerebellar_Hemisphere | PD |
| ENSG00000130787 | 12 | 2924 | rs10847864 | 0.999 | Brain_Cerebellum | PD |
| ENSG00000136243 | 7 | 2917 | rs858295 | 0.979 | Brain_Cerebellum | PD |
| ENSG00000145214 | 4 | 2924 | rs34311866 | 0.988 | Brain_Cerebellum | PD |
| ENSG00000004468 | 4 | 2924 | rs4698412 | 0.996 | Brain_Cortex | PD |
| ENSG00000004468 | 4 | 2885 | rs4698412 | 0.993 | Brain_Frontal_Cortex_BA9 | PD |
| ENSG00000136243 | 7 | 2887 | rs858295 | 0.979 | Brain_Frontal_Cortex_BA9 | PD |
| ENSG00000136235 | 7 | 2887 | rs858295 | 0.950 | Brain_Hippocampus | PD |
| ENSG00000226816 | 7 | 5072 | rs858295 | 0.970 | Brain_Hippocampus | PD |
| ENSG00000004468 | 4 | 5490 | rs4698412 | 0.990 | Brain_Hypothalamus | PD |
| ENSG00000136235 | 7 | 4876 | rs858295 | 0.950 | Brain_Hypothalamus | PD |
| ENSG00000004468 | 4 | 5190 | rs4698412 | 0.996 | Brain_Nucleus_accumbens_basal_ganglia | PD |
| ENSG00000103496 | 16 | 6228 | rs12934900 | 0.981 | Brain_Nucleus_accumbens_basal_ganglia | PD |
| ENSG00000198648 | 2 | 6234 | rs4613239 | 0.986 | Brain_Nucleus_accumbens_basal_ganglia | PD |
| ENSG00000103496 | 16 | 6166 | rs12934900 | 0.970 | Breast_Mammary_Tissue | PD |
| ENSG00000226816 | 7 | 6157 | rs858295 | 0.979 | Breast_Mammary_Tissue | PD |
| ENSG00000079459 | 8 | 6162 | rs7818035 | 0.980 | Esophagus_Gastroesophageal_Junction | PD |
| ENSG00000163687 | 3 | 6159 | rs4488803 | 0.998 | Esophagus_Gastroesophageal_Junction | PD |
| ENSG00000226816 | 7 | 6166 | rs858295 | 0.978 | Esophagus_Gastroesophageal_Junction | PD |
| ENSG00000108932 | 17 | 6166 | rs7213778 | 0.968 | Esophagus_Mucosa | PD |
| ENSG00000226816 | 7 | 5995 | rs858295 | 0.977 | Esophagus_Mucosa | PD |
| ENSG00000079459 | 8 | 4991 | rs7818035 | 0.985 | Esophagus_Muscularis | PD |
| ENSG00000136243 | 7 | 4995 | rs858295 | 0.983 | Esophagus_Muscularis | PD |
| ENSG00000167395 | 16 | 4996 | rs12934900 | 0.965 | Esophagus_Muscularis | PD |
| ENSG00000226816 | 7 | 4996 | rs858295 | 0.980 | Esophagus_Muscularis | PD |
| ENSG00000099377 | 16 | 4897 | rs12934900 | 0.951 | Liver | PD |
| ENSG00000152128 | 2 | 2838 | rs6741007 | 0.989 | Liver | PD |
| ENSG00000099377 | 16 | 5943 | rs12934900 | 0.965 | Nerve_Tibial | PD |
| ENSG00000103496 | 16 | 5943 | rs12934900 | 0.973 | Nerve_Tibial | PD |
| ENSG00000152128 | 2 | 5943 | rs6741007 | 0.995 | Nerve_Tibial | PD |
| ENSG00000188906 | 12 | 5897 | rs75505347 | 0.996 | Nerve_Tibial | PD |
| ENSG00000214846 | 4 | 5900 | rs4698412 | 0.994 | Nerve_Tibial | PD |
| ENSG00000226816 | 7 | 6299 | rs858295 | 0.978 | Nerve_Tibial | PD |
| ENSG00000117280 | 1 | 6201 | rs823106 | 0.952 | Pituitary | PD |
| ENSG00000188906 | 12 | 6201 | rs75505347 | 0.988 | Stomach | PD |
| ENSG00000226816 | 7 | 6201 | rs858295 | 0.977 | Stomach | PD |
| ENSG00000108823 | 17 | 6186 | rs847685 | 0.992 | Testis | PD |
| ENSG00000198648 | 2 | 6201 | rs4613239 | 0.993 | Testis | PD |
| ENSG00000071054 | 2 | 6201 | rs4851487 | 0.987 | Thyroid | PD |
| ENSG00000079459 | 8 | 6201 | rs7818035 | 0.977 | Thyroid | PD |
| ENSG00000099377 | 16 | 6201 | rs12934900 | 0.957 | Thyroid | PD |
| ENSG00000136243 | 7 | 6201 | rs858295 | 0.974 | Thyroid | PD |
| ENSG00000152128 | 2 | 6201 | rs6741007 | 0.987 | Thyroid | PD |
| ENSG00000243368 | 3 | 5225 | rs10513789 | 0.963 | Thyroid | PD |
| ENSG00000279196 | 16 | 2925 | rs12934900 | 0.976 | Thyroid | PD |

**Table S10.** Summary for pleiotropic genes of rheumatoid arthritis in 21 tissues

| Ensembl ID | Chromosome | No. SNPs | Lead SNP | PP H4 | Tissue | Disease |
| --- | --- | --- | --- | --- | --- | --- |
| ENSG00000054282 | 1 | 823 | rs10926994 | 0.978 | Adipose_Subcutaneous | RA |
| ENSG00000101017 | 20 | 823 | rs4239702 | 0.958 | Adipose_Subcutaneous | RA |
| ENSG00000103549 | 16 | 823 | rs2289442 | 0.990 | Adipose_Subcutaneous | RA |
| ENSG00000123411 | 12 | 823 | rs1873914 | 0.996 | Adipose_Subcutaneous | RA |
| ENSG00000134824 | 11 | 823 | rs968567 | 0.998 | Adipose_Subcutaneous | RA |
| ENSG00000136573 | 8 | 823 | rs2736340 | 0.987 | Adipose_Subcutaneous | RA |
| ENSG00000136689 | 2 | 1023 | rs17669228 | 0.952 | Adipose_Subcutaneous | RA |
| ENSG00000137806 | 15 | 1023 | rs1757457 | 0.963 | Adipose_Subcutaneous | RA |
| ENSG00000139531 | 12 | 1023 | rs1873914 | 0.996 | Adipose_Subcutaneous | RA |
| ENSG00000140368 | 15 | 1023 | rs4243046 | 0.974 | Adipose_Subcutaneous | RA |
| ENSG00000142606 | 1 | 1055 | rs2843401 | 0.983 | Adipose_Subcutaneous | RA |
| ENSG00000156858 | 16 | 940 | rs2289442 | 0.978 | Adipose_Subcutaneous | RA |
| ENSG00000160161 | 19 | 940 | rs2010506 | 0.952 | Adipose_Subcutaneous | RA |
| ENSG00000160856 | 1 | 883 | rs3761959 | 0.992 | Adipose_Subcutaneous | RA |
| ENSG00000172057 | 17 | 883 | rs9909593 | 0.978 | Adipose_Subcutaneous | RA |
| ENSG00000172840 | 16 | 883 | rs7187623 | 0.973 | Adipose_Subcutaneous | RA |
| ENSG00000173517 | 15 | 883 | rs4243046 | 0.983 | Adipose_Subcutaneous | RA |
| ENSG00000174428 | 7 | 989 | rs35735900 | 0.981 | Adipose_Subcutaneous | RA |
| ENSG00000178093 | 19 | 989 | rs2010506 | 0.965 | Adipose_Subcutaneous | RA |
| ENSG00000181240 | 19 | 989 | rs2305058 | 0.981 | Adipose_Subcutaneous | RA |
| ENSG00000185627 | 11 | 740 | rs1045288 | 0.968 | Adipose_Subcutaneous | RA |
| ENSG00000185651 | 22 | 475 | rs11089637 | 0.966 | Adipose_Subcutaneous | RA |
| ENSG00000196118 | 16 | 491 | rs2289442 | 0.992 | Adipose_Subcutaneous | RA |
| ENSG00000204581 | 2 | 491 | rs13405741 | 0.991 | Adipose_Subcutaneous | RA |
| ENSG00000215912 | 1 | 741 | rs2843401 | 0.975 | Adipose_Subcutaneous | RA |
| ENSG00000228037 | 1 | 750 | rs2843401 | 0.986 | Adipose_Subcutaneous | RA |
| ENSG00000236935 | 11 | 1009 | rs479777 | 0.972 | Adipose_Subcutaneous | RA |
| ENSG00000244682 | 1 | 1177 | rs7529425 | 0.997 | Adipose_Subcutaneous | RA |
| ENSG00000250067 | 19 | 1177 | rs2010506 | 0.957 | Adipose_Subcutaneous | RA |
| ENSG00000277290 | 11 | 1177 | rs1045288 | 0.966 | Adipose_Subcutaneous | RA |
| ENSG00000054282 | 1 | 1177 | rs10926994 | 0.980 | Adipose_Visceral_Omentum | RA |
| ENSG00000101017 | 20 | 1177 | rs4239702 | 0.989 | Adipose_Visceral_Omentum | RA |
| ENSG00000103549 | 16 | 1177 | rs2289442 | 0.992 | Adipose_Visceral_Omentum | RA |
| ENSG00000119013 | 2 | 1177 | rs16837131 | 0.966 | Adipose_Visceral_Omentum | RA |
| ENSG00000129354 | 19 | 1177 | rs12460655 | 0.999 | Adipose_Visceral_Omentum | RA |
| ENSG00000134824 | 11 | 1175 | rs968567 | 0.998 | Adipose_Visceral_Omentum | RA |
| ENSG00000134825 | 11 | 1177 | rs968567 | 0.976 | Adipose_Visceral_Omentum | RA |
| ENSG00000139531 | 12 | 1177 | rs1873914 | 0.996 | Adipose_Visceral_Omentum | RA |
| ENSG00000160856 | 1 | 1177 | rs3761959 | 0.991 | Adipose_Visceral_Omentum | RA |
| ENSG00000172057 | 17 | 1177 | rs9909593 | 0.963 | Adipose_Visceral_Omentum | RA |
| ENSG00000177963 | 11 | 1177 | rs1045288 | 0.962 | Adipose_Visceral_Omentum | RA |
| ENSG00000181240 | 19 | 1177 | rs2305058 | 0.981 | Adipose_Visceral_Omentum | RA |
| ENSG00000184110 | 16 | 1177 | rs7500321 | 0.979 | Adipose_Visceral_Omentum | RA |
| ENSG00000184602 | 16 | 1177 | rs4439779 | 0.993 | Adipose_Visceral_Omentum | RA |
| ENSG00000185627 | 11 | 1177 | rs1045288 | 0.969 | Adipose_Visceral_Omentum | RA |
| ENSG00000215912 | 1 | 1177 | rs2843401 | 0.962 | Adipose_Visceral_Omentum | RA |
| ENSG00000225217 | 1 | 491 | rs7529425 | 0.997 | Adipose_Visceral_Omentum | RA |
| ENSG00000234336 | 7 | 491 | rs917117 | 0.967 | Adipose_Visceral_Omentum | RA |
| ENSG00000236935 | 11 | 491 | rs479777 | 0.958 | Adipose_Visceral_Omentum | RA |
| ENSG00000244682 | 1 | 491 | rs7529425 | 0.991 | Adipose_Visceral_Omentum | RA |
| ENSG00000277290 | 11 | 491 | rs1045288 | 0.966 | Adipose_Visceral_Omentum | RA |
| ENSG00000100321 | 22 | 490 | rs137685 | 0.996 | Adrenal_Gland | RA |
| ENSG00000101017 | 20 | 491 | rs4239702 | 0.989 | Adrenal_Gland | RA |
| ENSG00000103549 | 16 | 491 | rs2289442 | 0.991 | Adrenal_Gland | RA |
| ENSG00000133103 | 13 | 491 | rs9603612 | 0.950 | Adrenal_Gland | RA |
| ENSG00000135506 | 12 | 491 | rs238516 | 0.966 | Adrenal_Gland | RA |
| ENSG00000139531 | 12 | 491 | rs1873914 | 0.991 | Adrenal_Gland | RA |
| ENSG00000142606 | 1 | 491 | rs2843401 | 0.971 | Adrenal_Gland | RA |
| ENSG00000161180 | 22 | 491 | rs11089637 | 0.982 | Adrenal_Gland | RA |
| ENSG00000178093 | 19 | 491 | rs2010506 | 0.969 | Adrenal_Gland | RA |
| ENSG00000181240 | 19 | 491 | rs2305058 | 0.981 | Adrenal_Gland | RA |
| ENSG00000184110 | 16 | 491 | rs7500321 | 0.952 | Adrenal_Gland | RA |
| ENSG00000185627 | 11 | 672 | rs1045288 | 0.967 | Adrenal_Gland | RA |
| ENSG00000196118 | 16 | 740 | rs2289442 | 0.972 | Adrenal_Gland | RA |
| ENSG00000196275 | 7 | 1151 | rs35735900 | 0.987 | Adrenal_Gland | RA |
| ENSG00000225217 | 1 | 844 | rs7529425 | 0.991 | Adrenal_Gland | RA |
| ENSG00000244682 | 1 | 850 | rs7529425 | 0.979 | Adrenal_Gland | RA |
| ENSG00000250067 | 19 | 684 | rs2010506 | 0.978 | Adrenal_Gland | RA |
| ENSG00000263001 | 7 | 907 | rs35735900 | 0.959 | Adrenal_Gland | RA |
| ENSG00000273890 | 12 | 936 | rs1873914 | 0.958 | Adrenal_Gland | RA |
| ENSG00000274425 | 19 | 936 | rs12460655 | 0.993 | Adrenal_Gland | RA |
| ENSG00000277290 | 11 | 959 | rs1045288 | 0.966 | Adrenal_Gland | RA |
| ENSG00000281920 | 2 | 957 | rs1858036 | 0.956 | Adrenal_Gland | RA |
| ENSG00000006459 | 7 | 834 | rs4626515 | 0.994 | Brain_Caudate_basal_ganglia | RA |
| ENSG00000054282 | 1 | 834 | rs10926994 | 0.978 | Brain_Caudate_basal_ganglia | RA |
| ENSG00000103549 | 16 | 1110 | rs2289442 | 0.991 | Brain_Caudate_basal_ganglia | RA |
| ENSG00000124140 | 20 | 1164 | rs4239702 | 0.967 | Brain_Caudate_basal_ganglia | RA |
| ENSG00000139531 | 12 | 839 | rs1873914 | 0.992 | Brain_Caudate_basal_ganglia | RA |
| ENSG00000166046 | 12 | 673 | rs2288268 | 0.962 | Brain_Caudate_basal_ganglia | RA |
| ENSG00000168811 | 3 | 673 | rs4680536 | 0.964 | Brain_Caudate_basal_ganglia | RA |
| ENSG00000173517 | 15 | 1142 | rs4243046 | 0.983 | Brain_Caudate_basal_ganglia | RA |
| ENSG00000177963 | 11 | 868 | rs1045288 | 0.961 | Brain_Caudate_basal_ganglia | RA |
| ENSG00000181240 | 19 | 868 | rs2305058 | 0.981 | Brain_Caudate_basal_ganglia | RA |
| ENSG00000184110 | 16 | 868 | rs7500321 | 0.984 | Brain_Caudate_basal_ganglia | RA |
| ENSG00000184602 | 16 | 868 | rs4439779 | 0.969 | Brain_Caudate_basal_ganglia | RA |
| ENSG00000185627 | 11 | 868 | rs1045288 | 0.959 | Brain_Caudate_basal_ganglia | RA |
| ENSG00000196118 | 16 | 868 | rs2289442 | 0.988 | Brain_Caudate_basal_ganglia | RA |
| ENSG00000254634 | 16 | 864 | rs4787495 | 0.983 | Brain_Caudate_basal_ganglia | RA |
| ENSG00000277290 | 11 | 755 | rs1045288 | 0.968 | Brain_Caudate_basal_ganglia | RA |
| ENSG00000101017 | 20 | 755 | rs4239702 | 0.953 | Brain_Cerebellar_Hemisphere | RA |
| ENSG00000103549 | 16 | 755 | rs2289442 | 0.992 | Brain_Cerebellar_Hemisphere | RA |
| ENSG00000125648 | 19 | 755 | rs2305058 | 0.970 | Brain_Cerebellar_Hemisphere | RA |
| ENSG00000173457 | 11 | 753 | rs479777 | 0.958 | Brain_Cerebellar_Hemisphere | RA |
| ENSG00000181240 | 19 | 1014 | rs2305058 | 0.981 | Brain_Cerebellar_Hemisphere | RA |
| ENSG00000183336 | 16 | 1033 | rs4787495 | 0.961 | Brain_Cerebellar_Hemisphere | RA |
| ENSG00000185627 | 11 | 1223 | rs1045288 | 0.968 | Brain_Cerebellar_Hemisphere | RA |
| ENSG00000215912 | 1 | 1223 | rs2843401 | 0.976 | Brain_Cerebellar_Hemisphere | RA |
| ENSG00000277072 | 7 | 1180 | rs35735900 | 0.969 | Brain_Cerebellar_Hemisphere | RA |
| ENSG00000277290 | 11 | 1180 | rs1045288 | 0.968 | Brain_Cerebellar_Hemisphere | RA |
| ENSG00000006459 | 7 | 836 | rs4626515 | 0.983 | Brain_Cerebellum | RA |
| ENSG00000103549 | 16 | 836 | rs2289442 | 0.991 | Brain_Cerebellum | RA |
| ENSG00000123297 | 12 | 836 | rs238516 | 0.960 | Brain_Cerebellum | RA |
| ENSG00000125648 | 19 | 836 | rs2305058 | 0.981 | Brain_Cerebellum | RA |
| ENSG00000133103 | 13 | 836 | rs9603612 | 0.978 | Brain_Cerebellum | RA |
| ENSG00000139531 | 12 | 836 | rs1873914 | 0.996 | Brain_Cerebellum | RA |
| ENSG00000157152 | 3 | 836 | rs305500 | 0.999 | Brain_Cerebellum | RA |
| ENSG00000162927 | 2 | 836 | rs34695944 | 0.994 | Brain_Cerebellum | RA |
| ENSG00000169203 | 16 | 836 | rs4787495 | 0.957 | Brain_Cerebellum | RA |
| ENSG00000181240 | 19 | 836 | rs2305058 | 0.981 | Brain_Cerebellum | RA |
| ENSG00000185627 | 11 | 836 | rs1045288 | 0.967 | Brain_Cerebellum | RA |
| ENSG00000187664 | 19 | 847 | rs2010506 | 0.983 | Brain_Cerebellum | RA |
| ENSG00000215912 | 1 | 847 | rs2843401 | 0.987 | Brain_Cerebellum | RA |
| ENSG00000261766 | 16 | 827 | rs7500321 | 0.951 | Brain_Cerebellum | RA |
| ENSG00000277290 | 11 | 827 | rs1045288 | 0.968 | Brain_Cerebellum | RA |
| ENSG00000006459 | 7 | 827 | rs4626515 | 0.995 | Brain_Cortex | RA |
| ENSG00000035141 | 2 | 827 | rs1986601 | 0.950 | Brain_Cortex | RA |
| ENSG00000099377 | 16 | 827 | rs2289442 | 0.990 | Brain_Cortex | RA |
| ENSG00000101017 | 20 | 1071 | rs4239702 | 0.992 | Brain_Cortex | RA |
| ENSG00000103549 | 16 | 1071 | rs2289442 | 0.990 | Brain_Cortex | RA |
| ENSG00000139531 | 12 | 851 | rs1873914 | 0.985 | Brain_Cortex | RA |
| ENSG00000159339 | 1 | 742 | rs1748041 | 0.972 | Brain_Cortex | RA |
| ENSG00000168243 | 1 | 742 | rs6429207 | 0.996 | Brain_Cortex | RA |
| ENSG00000177963 | 11 | 733 | rs1045288 | 0.964 | Brain_Cortex | RA |
| ENSG00000181240 | 19 | 678 | rs2305058 | 0.981 | Brain_Cortex | RA |
| ENSG00000183336 | 16 | 678 | rs4787495 | 0.982 | Brain_Cortex | RA |
| ENSG00000185627 | 11 | 678 | rs1045288 | 0.968 | Brain_Cortex | RA |
| ENSG00000196118 | 16 | 678 | rs2289442 | 0.991 | Brain_Cortex | RA |
| ENSG00000213658 | 16 | 678 | rs7500321 | 0.991 | Brain_Cortex | RA |
| ENSG00000260302 | 18 | 678 | rs478582 | 0.990 | Brain_Cortex | RA |
| ENSG00000274425 | 19 | 678 | rs12460655 | 0.997 | Brain_Cortex | RA |
| ENSG00000277290 | 11 | 678 | rs1045288 | 0.968 | Brain_Cortex | RA |
| ENSG00000006459 | 7 | 678 | rs4626515 | 0.995 | Brain_Frontal_Cortex_BA9 | RA |
| ENSG00000101017 | 20 | 676 | rs4239702 | 0.990 | Brain_Frontal_Cortex_BA9 | RA |
| ENSG00000140368 | 15 | 678 | rs4243046 | 0.983 | Brain_Frontal_Cortex_BA9 | RA |
| ENSG00000159339 | 1 | 678 | rs1748041 | 0.972 | Brain_Frontal_Cortex_BA9 | RA |
| ENSG00000177963 | 11 | 678 | rs1045288 | 0.958 | Brain_Frontal_Cortex_BA9 | RA |
| ENSG00000181240 | 19 | 678 | rs2305058 | 0.981 | Brain_Frontal_Cortex_BA9 | RA |
| ENSG00000183336 | 16 | 678 | rs4787495 | 0.973 | Brain_Frontal_Cortex_BA9 | RA |
| ENSG00000184602 | 16 | 678 | rs4439779 | 0.968 | Brain_Frontal_Cortex_BA9 | RA |
| ENSG00000185627 | 11 | 678 | rs1045288 | 0.966 | Brain_Frontal_Cortex_BA9 | RA |
| ENSG00000196118 | 16 | 678 | rs2289442 | 0.991 | Brain_Frontal_Cortex_BA9 | RA |
| ENSG00000213658 | 16 | 678 | rs7500321 | 0.989 | Brain_Frontal_Cortex_BA9 | RA |
| ENSG00000259914 | 16 | 678 | rs877920 | 0.957 | Brain_Frontal_Cortex_BA9 | RA |
| ENSG00000260788 | 16 | 678 | rs877920 | 0.990 | Brain_Frontal_Cortex_BA9 | RA |
| ENSG00000274425 | 19 | 637 | rs12460655 | 0.997 | Brain_Frontal_Cortex_BA9 | RA |
| ENSG00000277290 | 11 | 637 | rs1045288 | 0.968 | Brain_Frontal_Cortex_BA9 | RA |
| ENSG00000101017 | 20 | 796 | rs4239702 | 0.987 | Brain_Hippocampus | RA |
| ENSG00000139531 | 12 | 775 | rs1873914 | 0.986 | Brain_Hippocampus | RA |
| ENSG00000161179 | 22 | 775 | rs11089637 | 0.981 | Brain_Hippocampus | RA |
| ENSG00000173517 | 15 | 775 | rs4243046 | 0.968 | Brain_Hippocampus | RA |
| ENSG00000181240 | 19 | 775 | rs2305058 | 0.981 | Brain_Hippocampus | RA |
| ENSG00000185627 | 11 | 775 | rs1045288 | 0.968 | Brain_Hippocampus | RA |
| ENSG00000196118 | 16 | 775 | rs2289442 | 0.991 | Brain_Hippocampus | RA |
| ENSG00000236104 | 6 | 842 | rs1612904 | 0.979 | Brain_Hippocampus | RA |
| ENSG00000260788 | 16 | 907 | rs877920 | 0.977 | Brain_Hippocampus | RA |
| ENSG00000274425 | 19 | 907 | rs12460655 | 0.997 | Brain_Hippocampus | RA |
| ENSG00000277290 | 11 | 1291 | rs1045288 | 0.968 | Brain_Hippocampus | RA |
| ENSG00000101017 | 20 | 993 | rs4239702 | 0.991 | Brain_Hypothalamus | RA |
| ENSG00000125648 | 19 | 1107 | rs2305058 | 0.985 | Brain_Hypothalamus | RA |
| ENSG00000139531 | 12 | 476 | rs1873914 | 0.978 | Brain_Hypothalamus | RA |
| ENSG00000142606 | 1 | 476 | rs2843401 | 0.969 | Brain_Hypothalamus | RA |
| ENSG00000166046 | 12 | 477 | rs2288268 | 0.992 | Brain_Hypothalamus | RA |
| ENSG00000173517 | 15 | 483 | rs4243046 | 0.987 | Brain_Hypothalamus | RA |
| ENSG00000185627 | 11 | 1013 | rs1045288 | 0.967 | Brain_Hypothalamus | RA |
| ENSG00000196118 | 16 | 1013 | rs2289442 | 0.990 | Brain_Hypothalamus | RA |
| ENSG00000243321 | 3 | 740 | rs6799396 | 0.979 | Brain_Hypothalamus | RA |
| ENSG00000274425 | 19 | 740 | rs12460655 | 0.990 | Brain_Hypothalamus | RA |
| ENSG00000277290 | 11 | 936 | rs1045288 | 0.968 | Brain_Hypothalamus | RA |
| ENSG00000006459 | 7 | 936 | rs4626515 | 0.966 | Brain_Nucleus_accumbens_basal_ganglia | RA |
| ENSG00000103549 | 16 | 622 | rs2289442 | 0.983 | Brain_Nucleus_accumbens_basal_ganglia | RA |
| ENSG00000139531 | 12 | 622 | rs1873914 | 0.991 | Brain_Nucleus_accumbens_basal_ganglia | RA |
| ENSG00000142606 | 1 | 622 | rs2843401 | 0.950 | Brain_Nucleus_accumbens_basal_ganglia | RA |
| ENSG00000160161 | 19 | 774 | rs2010506 | 0.974 | Brain_Nucleus_accumbens_basal_ganglia | RA |
| ENSG00000169203 | 16 | 774 | rs4787495 | 0.959 | Brain_Nucleus_accumbens_basal_ganglia | RA |
| ENSG00000177951 | 11 | 1067 | rs1045288 | 0.962 | Brain_Nucleus_accumbens_basal_ganglia | RA |
| ENSG00000181240 | 19 | 1067 | rs2305058 | 0.981 | Brain_Nucleus_accumbens_basal_ganglia | RA |
| ENSG00000183336 | 16 | 1067 | rs4787495 | 0.984 | Brain_Nucleus_accumbens_basal_ganglia | RA |
| ENSG00000184602 | 16 | 1067 | rs4439779 | 0.978 | Brain_Nucleus_accumbens_basal_ganglia | RA |
| ENSG00000185627 | 11 | 1067 | rs1045288 | 0.968 | Brain_Nucleus_accumbens_basal_ganglia | RA |
| ENSG00000196118 | 16 | 732 | rs2289442 | 0.992 | Brain_Nucleus_accumbens_basal_ganglia | RA |
| ENSG00000215912 | 1 | 729 | rs2843401 | 0.982 | Brain_Nucleus_accumbens_basal_ganglia | RA |
| ENSG00000254634 | 16 | 724 | rs4787495 | 0.978 | Brain_Nucleus_accumbens_basal_ganglia | RA |
| ENSG00000260788 | 16 | 729 | rs877920 | 0.992 | Brain_Nucleus_accumbens_basal_ganglia | RA |
| ENSG00000277290 | 11 | 729 | rs1045288 | 0.968 | Brain_Nucleus_accumbens_basal_ganglia | RA |
| ENSG00000101017 | 20 | 779 | rs4239702 | 0.956 | Brain_Substantia_nigra | RA |
| ENSG00000103549 | 16 | 887 | rs2289442 | 0.988 | Brain_Substantia_nigra | RA |
| ENSG00000139531 | 12 | 822 | rs1873914 | 0.990 | Brain_Substantia_nigra | RA |
| ENSG00000161180 | 22 | 753 | rs11089637 | 0.980 | Brain_Substantia_nigra | RA |
| ENSG00000173517 | 15 | 792 | rs4243046 | 0.981 | Brain_Substantia_nigra | RA |
| ENSG00000183336 | 16 | 1107 | rs4787495 | 0.963 | Brain_Substantia_nigra | RA |
| ENSG00000185627 | 11 | 1107 | rs1045288 | 0.968 | Brain_Substantia_nigra | RA |
| ENSG00000277290 | 11 | 739 | rs1045288 | 0.968 | Brain_Substantia_nigra | RA |
| ENSG00000010030 | 6 | 1079 | rs879036 | 0.996 | Breast_Mammary_Tissue | RA |
| ENSG00000101017 | 20 | 897 | rs4239702 | 0.998 | Breast_Mammary_Tissue | RA |
| ENSG00000108559 | 17 | 897 | rs2641244 | 0.966 | Breast_Mammary_Tissue | RA |
| ENSG00000128604 | 7 | 897 | rs10488631 | 0.983 | Breast_Mammary_Tissue | RA |
| ENSG00000134824 | 11 | 1024 | rs968567 | 0.998 | Breast_Mammary_Tissue | RA |
| ENSG00000136573 | 8 | 1024 | rs2736340 | 0.968 | Breast_Mammary_Tissue | RA |
| ENSG00000139531 | 12 | 794 | rs1873914 | 0.992 | Breast_Mammary_Tissue | RA |
| ENSG00000160161 | 19 | 384 | rs2010506 | 0.954 | Breast_Mammary_Tissue | RA |
| ENSG00000168243 | 1 | 384 | rs6429207 | 0.958 | Breast_Mammary_Tissue | RA |
| ENSG00000173517 | 15 | 384 | rs4243046 | 0.983 | Breast_Mammary_Tissue | RA |
| ENSG00000177963 | 11 | 384 | rs1045288 | 0.966 | Breast_Mammary_Tissue | RA |
| ENSG00000181240 | 19 | 384 | rs2305058 | 0.981 | Breast_Mammary_Tissue | RA |
| ENSG00000181625 | 16 | 384 | rs4787495 | 0.981 | Breast_Mammary_Tissue | RA |
| ENSG00000184602 | 16 | 388 | rs4439779 | 0.995 | Breast_Mammary_Tissue | RA |
| ENSG00000185627 | 11 | 454 | rs1045288 | 0.969 | Breast_Mammary_Tissue | RA |
| ENSG00000205108 | 9 | 708 | rs11574914 | 0.995 | Breast_Mammary_Tissue | RA |
| ENSG00000215912 | 1 | 708 | rs2843401 | 0.982 | Breast_Mammary_Tissue | RA |
| ENSG00000225217 | 1 | 1112 | rs7529425 | 0.955 | Breast_Mammary_Tissue | RA |
| ENSG00000236935 | 11 | 714 | rs479777 | 0.957 | Breast_Mammary_Tissue | RA |
| ENSG00000244682 | 1 | 727 | rs7529425 | 0.957 | Breast_Mammary_Tissue | RA |
| ENSG00000246575 | 2 | 779 | rs10460587 | 0.966 | Breast_Mammary_Tissue | RA |
| ENSG00000250067 | 19 | 695 | rs2010506 | 0.963 | Breast_Mammary_Tissue | RA |
| ENSG00000259038 | 14 | 695 | rs911263 | 0.955 | Breast_Mammary_Tissue | RA |
| ENSG00000277290 | 11 | 710 | rs1045288 | 0.966 | Breast_Mammary_Tissue | RA |
| ENSG00000134824 | 11 | 710 | rs968567 | 0.998 | Colon_Transverse | RA |
| ENSG00000139531 | 12 | 710 | rs1873914 | 0.996 | Colon_Transverse | RA |
| ENSG00000173517 | 15 | 710 | rs4243046 | 0.983 | Colon_Transverse | RA |
| ENSG00000181240 | 19 | 710 | rs2305058 | 0.981 | Colon_Transverse | RA |
| ENSG00000184110 | 16 | 710 | rs7500321 | 0.956 | Colon_Transverse | RA |
| ENSG00000184602 | 16 | 710 | rs4439779 | 0.981 | Colon_Transverse | RA |
| ENSG00000185627 | 11 | 710 | rs1045288 | 0.970 | Colon_Transverse | RA |
| ENSG00000213658 | 16 | 710 | rs7500321 | 0.986 | Colon_Transverse | RA |
| ENSG00000277290 | 11 | 710 | rs1045288 | 0.966 | Colon_Transverse | RA |
| ENSG00000064012 | 2 | 710 | rs16837131 | 0.996 | Esophagus_Gastroesophageal_Junction | RA |
| ENSG00000065989 | 19 | 710 | rs12460655 | 0.979 | Esophagus_Gastroesophageal_Junction | RA |
| ENSG00000101017 | 20 | 411 | rs4239702 | 0.993 | Esophagus_Gastroesophageal_Junction | RA |
| ENSG00000103549 | 16 | 1035 | rs2289442 | 0.986 | Esophagus_Gastroesophageal_Junction | RA |
| ENSG00000119403 | 9 | 960 | rs10818482 | 0.984 | Esophagus_Gastroesophageal_Junction | RA |
| ENSG00000128604 | 7 | 960 | rs10488631 | 0.992 | Esophagus_Gastroesophageal_Junction | RA |
| ENSG00000134824 | 11 | 492 | rs968567 | 0.998 | Esophagus_Gastroesophageal_Junction | RA |
| ENSG00000135506 | 12 | 492 | rs238516 | 0.988 | Esophagus_Gastroesophageal_Junction | RA |
| ENSG00000139531 | 12 | 492 | rs1873914 | 0.996 | Esophagus_Gastroesophageal_Junction | RA |
| ENSG00000142606 | 1 | 492 | rs2843401 | 0.983 | Esophagus_Gastroesophageal_Junction | RA |
| ENSG00000156858 | 16 | 492 | rs2289442 | 0.969 | Esophagus_Gastroesophageal_Junction | RA |
| ENSG00000156873 | 16 | 492 | rs2289442 | 0.983 | Esophagus_Gastroesophageal_Junction | RA |
| ENSG00000173517 | 15 | 492 | rs4243046 | 0.973 | Esophagus_Gastroesophageal_Junction | RA |
| ENSG00000175354 | 18 | 492 | rs478582 | 0.979 | Esophagus_Gastroesophageal_Junction | RA |
| ENSG00000181240 | 19 | 626 | rs2305058 | 0.981 | Esophagus_Gastroesophageal_Junction | RA |
| ENSG00000182004 | 1 | 626 | rs11240609 | 0.958 | Esophagus_Gastroesophageal_Junction | RA |
| ENSG00000184110 | 16 | 626 | rs7500321 | 0.976 | Esophagus_Gastroesophageal_Junction | RA |
| ENSG00000185627 | 11 | 626 | rs1045288 | 0.967 | Esophagus_Gastroesophageal_Junction | RA |
| ENSG00000213658 | 16 | 626 | rs7500321 | 0.980 | Esophagus_Gastroesophageal_Junction | RA |
| ENSG00000215912 | 1 | 432 | rs2843401 | 0.979 | Esophagus_Gastroesophageal_Junction | RA |
| ENSG00000225217 | 1 | 432 | rs7529425 | 0.995 | Esophagus_Gastroesophageal_Junction | RA |
| ENSG00000234336 | 7 | 871 | rs917117 | 0.995 | Esophagus_Gastroesophageal_Junction | RA |
| ENSG00000244682 | 1 | 871 | rs7529425 | 0.995 | Esophagus_Gastroesophageal_Junction | RA |
| ENSG00000277290 | 11 | 871 | rs1045288 | 0.966 | Esophagus_Gastroesophageal_Junction | RA |
| ENSG00000054282 | 1 | 871 | rs10926994 | 0.966 | Esophagus_Mucosa | RA |
| ENSG00000101017 | 20 | 871 | rs4239702 | 0.985 | Esophagus_Mucosa | RA |
| ENSG00000120217 | 9 | 871 | rs911760 | 0.969 | Esophagus_Mucosa | RA |
| ENSG00000120659 | 13 | 871 | rs7316953 | 0.968 | Esophagus_Mucosa | RA |
| ENSG00000123411 | 12 | 871 | rs1873914 | 0.994 | Esophagus_Mucosa | RA |
| ENSG00000134824 | 11 | 871 | rs968567 | 0.998 | Esophagus_Mucosa | RA |
| ENSG00000139531 | 12 | 871 | rs1873914 | 0.996 | Esophagus_Mucosa | RA |
| ENSG00000142599 | 1 | 871 | rs301802 | 0.955 | Esophagus_Mucosa | RA |
| ENSG00000143811 | 1 | 871 | rs14868 | 0.961 | Esophagus_Mucosa | RA |
| ENSG00000152291 | 2 | 871 | rs10460587 | 0.967 | Esophagus_Mucosa | RA |
| ENSG00000160712 | 1 | 871 | rs4129267 | 0.961 | Esophagus_Mucosa | RA |
| ENSG00000160856 | 1 | 871 | rs3761959 | 0.993 | Esophagus_Mucosa | RA |
| ENSG00000161180 | 22 | 871 | rs11089637 | 0.954 | Esophagus_Mucosa | RA |
| ENSG00000162924 | 2 | 871 | rs34695944 | 0.957 | Esophagus_Mucosa | RA |
| ENSG00000167987 | 11 | 871 | rs968567 | 0.955 | Esophagus_Mucosa | RA |
| ENSG00000172828 | 16 | 871 | rs7187623 | 0.969 | Esophagus_Mucosa | RA |
| ENSG00000172831 | 16 | 871 | rs7187623 | 0.963 | Esophagus_Mucosa | RA |
| ENSG00000181240 | 19 | 871 | rs2305058 | 0.981 | Esophagus_Mucosa | RA |
| ENSG00000185627 | 11 | 871 | rs1045288 | 0.970 | Esophagus_Mucosa | RA |
| ENSG00000185651 | 22 | 372 | rs11089637 | 0.952 | Esophagus_Mucosa | RA |
| ENSG00000196118 | 16 | 372 | rs2289442 | 0.984 | Esophagus_Mucosa | RA |
| ENSG00000213658 | 16 | 1142 | rs7500321 | 0.986 | Esophagus_Mucosa | RA |
| ENSG00000215912 | 1 | 815 | rs2843401 | 0.972 | Esophagus_Mucosa | RA |
| ENSG00000244682 | 1 | 372 | rs7529425 | 0.995 | Esophagus_Mucosa | RA |
| ENSG00000246575 | 2 | 372 | rs10460587 | 0.969 | Esophagus_Mucosa | RA |
| ENSG00000250067 | 19 | 372 | rs2010506 | 0.959 | Esophagus_Mucosa | RA |
| ENSG00000274425 | 19 | 372 | rs12460655 | 0.982 | Esophagus_Mucosa | RA |
| ENSG00000277290 | 11 | 372 | rs1045288 | 0.966 | Esophagus_Mucosa | RA |
| ENSG00000064012 | 2 | 372 | rs16837131 | 0.998 | Esophagus_Muscularis | RA |
| ENSG00000100599 | 14 | 372 | rs11627032 | 0.969 | Esophagus_Muscularis | RA |
| ENSG00000101017 | 20 | 514 | rs4239702 | 0.992 | Esophagus_Muscularis | RA |
| ENSG00000103549 | 16 | 514 | rs2289442 | 0.992 | Esophagus_Muscularis | RA |
| ENSG00000119403 | 9 | 514 | rs10818482 | 0.984 | Esophagus_Muscularis | RA |
| ENSG00000128604 | 7 | 514 | rs10488631 | 0.969 | Esophagus_Muscularis | RA |
| ENSG00000134824 | 11 | 514 | rs968567 | 0.998 | Esophagus_Muscularis | RA |
| ENSG00000134825 | 11 | 514 | rs968567 | 0.964 | Esophagus_Muscularis | RA |
| ENSG00000139531 | 12 | 514 | rs1873914 | 0.997 | Esophagus_Muscularis | RA |
| ENSG00000154124 | 5 | 514 | rs25986 | 0.978 | Esophagus_Muscularis | RA |
| ENSG00000160712 | 1 | 1307 | rs4129267 | 0.957 | Esophagus_Muscularis | RA |
| ENSG00000169203 | 16 | 1307 | rs4787495 | 0.980 | Esophagus_Muscularis | RA |
| ENSG00000169682 | 16 | 1307 | rs7500321 | 0.990 | Esophagus_Muscularis | RA |
| ENSG00000173517 | 15 | 1307 | rs4243046 | 0.975 | Esophagus_Muscularis | RA |
| ENSG00000178093 | 19 | 1307 | rs2010506 | 0.970 | Esophagus_Muscularis | RA |
| ENSG00000181240 | 19 | 1307 | rs2305058 | 0.981 | Esophagus_Muscularis | RA |
| ENSG00000184110 | 16 | 1307 | rs7500321 | 0.967 | Esophagus_Muscularis | RA |
| ENSG00000185627 | 11 | 503 | rs1045288 | 0.968 | Esophagus_Muscularis | RA |
| ENSG00000185651 | 22 | 503 | rs11089637 | 0.967 | Esophagus_Muscularis | RA |
| ENSG00000205609 | 16 | 503 | rs7500321 | 0.965 | Esophagus_Muscularis | RA |
| ENSG00000213658 | 16 | 503 | rs7500321 | 0.991 | Esophagus_Muscularis | RA |
| ENSG00000215912 | 1 | 503 | rs2843401 | 0.975 | Esophagus_Muscularis | RA |
| ENSG00000220201 | 19 | 503 | rs12460655 | 0.992 | Esophagus_Muscularis | RA |
| ENSG00000225217 | 1 | 503 | rs7529425 | 0.955 | Esophagus_Muscularis | RA |
| ENSG00000244682 | 1 | 503 | rs7529425 | 0.996 | Esophagus_Muscularis | RA |
| ENSG00000246575 | 2 | 503 | rs10460587 | 0.982 | Esophagus_Muscularis | RA |
| ENSG00000267053 | 19 | 503 | rs6510528 | 0.968 | Esophagus_Muscularis | RA |
| ENSG00000277290 | 11 | 503 | rs1045288 | 0.966 | Esophagus_Muscularis | RA |
| ENSG00000103549 | 16 | 503 | rs2289442 | 0.989 | Liver | RA |
| ENSG00000133466 | 22 | 503 | rs6000603 | 0.951 | Liver | RA |
| ENSG00000137806 | 15 | 503 | rs1757457 | 0.973 | Liver | RA |
| ENSG00000139531 | 12 | 503 | rs1873914 | 0.990 | Liver | RA |
| ENSG00000157873 | 1 | 503 | rs2843401 | 0.982 | Liver | RA |
| ENSG00000160856 | 1 | 503 | rs3761959 | 0.992 | Liver | RA |
| ENSG00000169203 | 16 | 503 | rs4787495 | 0.973 | Liver | RA |
| ENSG00000173517 | 15 | 503 | rs4243046 | 0.975 | Liver | RA |
| ENSG00000181240 | 19 | 503 | rs2305058 | 0.981 | Liver | RA |
| ENSG00000185627 | 11 | 503 | rs1045288 | 0.962 | Liver | RA |
| ENSG00000225217 | 1 | 503 | rs7529425 | 0.979 | Liver | RA |
| ENSG00000244682 | 1 | 503 | rs7529425 | 0.992 | Liver | RA |
| ENSG00000250067 | 19 | 769 | rs2010506 | 0.983 | Liver | RA |
| ENSG00000277290 | 11 | 769 | rs1045288 | 0.968 | Liver | RA |
| ENSG00000035141 | 2 | 769 | rs1986601 | 0.957 | Muscle_Skeletal | RA |
| ENSG00000099385 | 16 | 769 | rs2289442 | 0.965 | Muscle_Skeletal | RA |
| ENSG00000101017 | 20 | 769 | rs4239702 | 0.973 | Muscle_Skeletal | RA |
| ENSG00000105705 | 19 | 709 | rs2010506 | 0.990 | Muscle_Skeletal | RA |
| ENSG00000125648 | 19 | 689 | rs2305058 | 0.981 | Muscle_Skeletal | RA |
| ENSG00000128342 | 22 | 491 | rs713875 | 0.979 | Muscle_Skeletal | RA |
| ENSG00000129744 | 11 | 491 | rs10834744 | 0.964 | Muscle_Skeletal | RA |
| ENSG00000129749 | 11 | 491 | rs10834744 | 0.964 | Muscle_Skeletal | RA |
| ENSG00000133466 | 22 | 491 | rs6000603 | 0.972 | Muscle_Skeletal | RA |
| ENSG00000134824 | 11 | 491 | rs968567 | 0.963 | Muscle_Skeletal | RA |
| ENSG00000135506 | 12 | 491 | rs238516 | 0.984 | Muscle_Skeletal | RA |
| ENSG00000137825 | 15 | 491 | rs1757457 | 0.956 | Muscle_Skeletal | RA |
| ENSG00000139531 | 12 | 491 | rs1873914 | 0.986 | Muscle_Skeletal | RA |
| ENSG00000155974 | 12 | 491 | rs11176611 | 0.971 | Muscle_Skeletal | RA |
| ENSG00000167987 | 11 | 491 | rs968567 | 0.969 | Muscle_Skeletal | RA |
| ENSG00000169203 | 16 | 491 | rs4787495 | 0.977 | Muscle_Skeletal | RA |
| ENSG00000169957 | 16 | 491 | rs2289442 | 0.981 | Muscle_Skeletal | RA |
| ENSG00000173517 | 15 | 491 | rs4243046 | 0.952 | Muscle_Skeletal | RA |
| ENSG00000181240 | 19 | 404 | rs2305058 | 0.981 | Muscle_Skeletal | RA |
| ENSG00000183336 | 16 | 432 | rs4787495 | 0.952 | Muscle_Skeletal | RA |
| ENSG00000184110 | 16 | 1036 | rs7500321 | 0.984 | Muscle_Skeletal | RA |
| ENSG00000185651 | 22 | 611 | rs11089637 | 0.976 | Muscle_Skeletal | RA |
| ENSG00000200201 | 11 | 611 | rs10834744 | 0.964 | Muscle_Skeletal | RA |
| ENSG00000213658 | 16 | 741 | rs7500321 | 0.988 | Muscle_Skeletal | RA |
| ENSG00000244682 | 1 | 741 | rs7529425 | 0.987 | Muscle_Skeletal | RA |
| ENSG00000262420 | 16 | 586 | rs4439779 | 0.991 | Muscle_Skeletal | RA |
| ENSG00000277290 | 11 | 389 | rs1045288 | 0.968 | Muscle_Skeletal | RA |
| ENSG00000006459 | 7 | 389 | rs4626515 | 0.991 | Nerve_Tibial | RA |
| ENSG00000010030 | 6 | 389 | rs879036 | 0.999 | Nerve_Tibial | RA |
| ENSG00000015153 | 12 | 389 | rs7954523 | 0.964 | Nerve_Tibial | RA |
| ENSG00000064012 | 2 | 389 | rs16837131 | 1.000 | Nerve_Tibial | RA |
| ENSG00000101017 | 20 | 389 | rs4239702 | 0.992 | Nerve_Tibial | RA |
| ENSG00000103549 | 16 | 389 | rs2289442 | 0.991 | Nerve_Tibial | RA |
| ENSG00000115998 | 2 | 389 | rs1986601 | 0.989 | Nerve_Tibial | RA |
| ENSG00000134824 | 11 | 389 | rs968567 | 0.998 | Nerve_Tibial | RA |
| ENSG00000135506 | 12 | 846 | rs238516 | 0.976 | Nerve_Tibial | RA |
| ENSG00000139531 | 12 | 846 | rs1873914 | 0.996 | Nerve_Tibial | RA |
| ENSG00000156860 | 16 | 846 | rs2289442 | 0.991 | Nerve_Tibial | RA |
| ENSG00000173517 | 15 | 846 | rs4243046 | 0.980 | Nerve_Tibial | RA |
| ENSG00000174640 | 3 | 846 | rs4854790 | 0.953 | Nerve_Tibial | RA |
| ENSG00000178093 | 19 | 846 | rs2010506 | 0.979 | Nerve_Tibial | RA |
| ENSG00000178188 | 16 | 846 | rs7500321 | 0.964 | Nerve_Tibial | RA |
| ENSG00000181240 | 19 | 846 | rs2305058 | 0.981 | Nerve_Tibial | RA |
| ENSG00000184110 | 16 | 846 | rs7500321 | 0.987 | Nerve_Tibial | RA |
| ENSG00000185627 | 11 | 846 | rs1045288 | 0.967 | Nerve_Tibial | RA |
| ENSG00000196118 | 16 | 846 | rs2289442 | 0.991 | Nerve_Tibial | RA |
| ENSG00000205108 | 9 | 846 | rs11574914 | 0.992 | Nerve_Tibial | RA |
| ENSG00000215912 | 1 | 846 | rs2843401 | 0.977 | Nerve_Tibial | RA |
| ENSG00000234336 | 7 | 753 | rs917117 | 0.989 | Nerve_Tibial | RA |
| ENSG00000246575 | 2 | 881 | rs10460587 | 0.975 | Nerve_Tibial | RA |
| ENSG00000277290 | 11 | 1071 | rs1045288 | 0.967 | Nerve_Tibial | RA |
| ENSG00000010030 | 6 | 1071 | rs879036 | 0.996 | Pituitary | RA |
| ENSG00000101017 | 20 | 1071 | rs4239702 | 0.985 | Pituitary | RA |
| ENSG00000125648 | 19 | 1071 | rs2305058 | 0.951 | Pituitary | RA |
| ENSG00000157152 | 3 | 1071 | rs305500 | 0.999 | Pituitary | RA |
| ENSG00000161180 | 22 | 1071 | rs11089637 | 0.993 | Pituitary | RA |
| ENSG00000173457 | 11 | 774 | rs479777 | 0.961 | Pituitary | RA |
| ENSG00000177963 | 11 | 750 | rs1045288 | 0.968 | Pituitary | RA |
| ENSG00000178188 | 16 | 1192 | rs7500321 | 0.956 | Pituitary | RA |
| ENSG00000181240 | 19 | 1192 | rs2305058 | 0.981 | Pituitary | RA |
| ENSG00000183336 | 16 | 1192 | rs4787495 | 0.973 | Pituitary | RA |
| ENSG00000185627 | 11 | 1192 | rs1045288 | 0.968 | Pituitary | RA |
| ENSG00000196118 | 16 | 948 | rs2289442 | 0.992 | Pituitary | RA |
| ENSG00000204581 | 2 | 2110 | rs13405741 | 0.999 | Pituitary | RA |
| ENSG00000246575 | 2 | 692 | rs10460587 | 0.970 | Pituitary | RA |
| ENSG00000277290 | 11 | 692 | rs1045288 | 0.968 | Pituitary | RA |
| ENSG00000101017 | 20 | 692 | rs4239702 | 0.996 | Stomach | RA |
| ENSG00000103549 | 16 | 764 | rs2289442 | 0.956 | Stomach | RA |
| ENSG00000134824 | 11 | 975 | rs968567 | 0.998 | Stomach | RA |
| ENSG00000139531 | 12 | 1069 | rs1873914 | 0.994 | Stomach | RA |
| ENSG00000152291 | 2 | 1069 | rs10460587 | 0.967 | Stomach | RA |
| ENSG00000167987 | 11 | 1069 | rs968567 | 0.970 | Stomach | RA |
| ENSG00000169203 | 16 | 1069 | rs4787495 | 0.977 | Stomach | RA |
| ENSG00000181240 | 19 | 1069 | rs2305058 | 0.981 | Stomach | RA |
| ENSG00000185627 | 11 | 1069 | rs1045288 | 0.968 | Stomach | RA |
| ENSG00000213658 | 16 | 1069 | rs7500321 | 0.989 | Stomach | RA |
| ENSG00000215912 | 1 | 1069 | rs2843401 | 0.974 | Stomach | RA |
| ENSG00000244682 | 1 | 1069 | rs7529425 | 0.991 | Stomach | RA |
| ENSG00000246575 | 2 | 1069 | rs10460587 | 0.975 | Stomach | RA |
| ENSG00000250067 | 19 | 1069 | rs2010506 | 0.967 | Stomach | RA |
| ENSG00000259345 | 15 | 911 | rs7170151 | 0.977 | Stomach | RA |
| ENSG00000277290 | 11 | 911 | rs1045288 | 0.966 | Stomach | RA |
| ENSG00000100316 | 22 | 911 | rs137685 | 0.997 | Testis | RA |
| ENSG00000101017 | 20 | 911 | rs4239702 | 0.991 | Testis | RA |
| ENSG00000103549 | 16 | 911 | rs2289442 | 0.973 | Testis | RA |
| ENSG00000105810 | 7 | 911 | rs8179 | 0.984 | Testis | RA |
| ENSG00000115459 | 2 | 911 | rs10460587 | 0.985 | Testis | RA |
| ENSG00000125648 | 19 | 623 | rs2305058 | 0.970 | Testis | RA |
| ENSG00000128604 | 7 | 623 | rs10488631 | 0.957 | Testis | RA |
| ENSG00000135506 | 12 | 623 | rs238516 | 0.971 | Testis | RA |
| ENSG00000139531 | 12 | 623 | rs1873914 | 0.997 | Testis | RA |
| ENSG00000163239 | 1 | 623 | rs4129267 | 0.953 | Testis | RA |
| ENSG00000163599 | 2 | 623 | rs3087243 | 0.996 | Testis | RA |
| ENSG00000172575 | 15 | 623 | rs7170151 | 0.985 | Testis | RA |
| ENSG00000177951 | 11 | 370 | rs1045288 | 0.969 | Testis | RA |
| ENSG00000181240 | 19 | 370 | rs2305058 | 0.981 | Testis | RA |
| ENSG00000185627 | 11 | 1010 | rs1045288 | 0.968 | Testis | RA |
| ENSG00000185651 | 22 | 1156 | rs11089637 | 0.969 | Testis | RA |
| ENSG00000186075 | 17 | 2249 | rs9909593 | 0.978 | Testis | RA |
| ENSG00000196118 | 16 | 940 | rs2289442 | 0.986 | Testis | RA |
| ENSG00000196296 | 16 | 2247 | rs7500321 | 0.976 | Testis | RA |
| ENSG00000213658 | 16 | 2247 | rs7500321 | 0.977 | Testis | RA |
| ENSG00000215912 | 1 | 2247 | rs2843401 | 0.952 | Testis | RA |
| ENSG00000228013 | 1 | 431 | rs4129267 | 0.982 | Testis | RA |
| ENSG00000235101 | 1 | 1286 | rs7529425 | 0.966 | Testis | RA |
| ENSG00000275106 | 7 | 1286 | rs10488631 | 0.977 | Testis | RA |
| ENSG00000277290 | 11 | 396 | rs1045288 | 0.968 | Testis | RA |
| ENSG00000010030 | 6 | 713 | rs879036 | 0.999 | Thyroid | RA |
| ENSG00000099385 | 16 | 670 | rs2289442 | 0.973 | Thyroid | RA |
| ENSG00000101017 | 20 | 754 | rs4239702 | 0.991 | Thyroid | RA |
| ENSG00000103549 | 16 | 754 | rs2289442 | 0.992 | Thyroid | RA |
| ENSG00000111052 | 12 | 754 | rs11114692 | 0.956 | Thyroid | RA |
| ENSG00000111058 | 12 | 754 | rs11114692 | 0.958 | Thyroid | RA |
| ENSG00000111540 | 12 | 754 | rs1873914 | 0.994 | Thyroid | RA |
| ENSG00000115998 | 2 | 754 | rs1986601 | 0.957 | Thyroid | RA |
| ENSG00000118985 | 5 | 755 | rs3777165 | 0.951 | Thyroid | RA |
| ENSG00000134824 | 11 | 400 | rs968567 | 0.998 | Thyroid | RA |
| ENSG00000139531 | 12 | 508 | rs1873914 | 0.997 | Thyroid | RA |
| ENSG00000142606 | 1 | 508 | rs2843401 | 0.977 | Thyroid | RA |
| ENSG00000153066 | 16 | 508 | rs4439779 | 0.955 | Thyroid | RA |
| ENSG00000157873 | 1 | 508 | rs2843401 | 0.978 | Thyroid | RA |
| ENSG00000160856 | 1 | 508 | rs3761959 | 0.989 | Thyroid | RA |
| ENSG00000162896 | 1 | 508 | rs2275529 | 0.984 | Thyroid | RA |
| ENSG00000166589 | 16 | 508 | rs7187623 | 0.965 | Thyroid | RA |
| ENSG00000166949 | 15 | 508 | rs17293632 | 0.985 | Thyroid | RA |
| ENSG00000175354 | 18 | 508 | rs478582 | 0.982 | Thyroid | RA |
| ENSG00000178093 | 19 | 508 | rs2010506 | 0.953 | Thyroid | RA |
| ENSG00000181240 | 19 | 508 | rs2305058 | 0.981 | Thyroid | RA |
| ENSG00000181625 | 16 | 508 | rs4787495 | 0.982 | Thyroid | RA |
| ENSG00000182795 | 1 | 508 | rs2275529 | 0.963 | Thyroid | RA |
| ENSG00000184602 | 16 | 508 | rs4439779 | 0.989 | Thyroid | RA |
| ENSG00000185627 | 11 | 508 | rs1045288 | 0.966 | Thyroid | RA |
| ENSG00000196118 | 16 | 508 | rs2289442 | 0.991 | Thyroid | RA |
| ENSG00000215912 | 1 | 508 | rs2843401 | 0.959 | Thyroid | RA |
| ENSG00000224727 | 1 | 508 | rs10926994 | 0.985 | Thyroid | RA |
| ENSG00000234336 | 7 | 508 | rs917117 | 0.968 | Thyroid | RA |
| ENSG00000243069 | 3 | 508 | rs1727946 | 0.953 | Thyroid | RA |
| ENSG00000244682 | 1 | 508 | rs7529425 | 0.997 | Thyroid | RA |
| ENSG00000246575 | 2 | 508 | rs10460587 | 0.989 | Thyroid | RA |
| ENSG00000250067 | 19 | 508 | rs2010506 | 0.953 | Thyroid | RA |
| ENSG00000262420 | 16 | 508 | rs4439779 | 0.990 | Thyroid | RA |
| ENSG00000277290 | 11 | 1072 | rs1045288 | 0.966 | Thyroid | RA |

| **Table S11.** Coloc results of pleiotropic genes shared by RA and PD in 21 tissues | | | | | | | |
| --- | --- | --- | --- | --- | --- | --- | --- |
| Gene | Ensembl ID | Chromosome | No. SNPs | Lead SNP | PP H4 of Genes | Tissue | Disease |
| HSD3B7 | ENSG00000099377 | 16 | 6259 | rs12934900 | 0.959 | Adipose_Subcutaneous | PD |
| HSD3B7 | ENSG00000099377 | 16 | 5833 | rs12934900 | 0.952 | Adrenal_Gland | PD |
| HSD3B7 | ENSG00000099377 | 16 | 4897 | rs12934900 | 0.951 | Liver | PD |
| HSD3B7 | ENSG00000099377 | 16 | 5943 | rs12934900 | 0.965 | Nerve_Tibial | PD |
| HSD3B7 | ENSG00000099377 | 16 | 6201 | rs12934900 | 0.957 | Thyroid | PD |
| HSD3B7 | ENSG00000099377 | 16 | 827 | rs2289442 | 0.990 | Brain_Cortex | RA |

**Table S12.** Descriptive characteristics of included GWAS datasets

| Name | N Cases | N Control | Database source | GWAS ID | OA/NOA | Publication date |
| --- | --- | --- | --- | --- | --- | --- |
| Osteoarthritis | 77052 | 378169 | GWAS catalog | GCST007093 | OA | 2019 |
| Rheumatoid arthritis | 22350 | 74823 | GWAS catalog | GCST90132223 | OA | 2022 |
| Alzheimer's disease | 25392 | 276086 | GWAS catalog | GCST90027158 | OA | 2022 |
| Parkinson's disease | 33674 | 449056 | IEU GWAS database | ieu-b-7 | OA | 2019 |
| Multiple sclerosis | 47429 | 68374 | IEU GWAS database | ieu-b-18 | OA | 2019 |

| **Table S13.** Details of primary antibodies used in this study | | |
| --- | --- | --- |
| Target Protein | Manufacturer | Catalog Number |
| RNF40 | Proteintech | 84058-4-RR |
| Tyrosine Hydroxylase (TH) | Proteintech | 25859-1-AP |
| Lonized calcium-binding adaptor molecule 1 (Iba1) | FUJIFILM Wako | 019-19741 |
| Glial Fibrillary Acidic Protein (GFAP) | Proteintech | 16825-1-AP |
